# Plasma metabolomics reveals disrupted response and recovery following maximal exercise in myalgic encephalomyelitis/chronic fatigue syndrome

**DOI:** 10.1172/jci.insight.157621

**Published:** 2022-05-09

**Authors:** Arnaud Germain, Ludovic Giloteaux, Geoffrey E. Moore, Susan M. Levine, John K. Chia, Betsy A. Keller, Jared Stevens, Carl J. Franconi, Xiangling Mao, Dikoma C. Shungu, Andrew Grimson, Maureen R. Hanson

**Affiliations:** 1 Department of Molecular Biology and Genetics, Cornell University, Ithaca, New York, USA.; 2 Department of Exercise Science and Athletic Training, Ithaca College, Ithaca, New York, USA.; 3 EVMED Research, Torrance, California, USA.; 4 Workwell Foundation, Ripon, California, USA.; 5 Department of Neuroradiology, Weill Cornell Medical College, New York, New York, USA.

**Keywords:** Metabolism, Amino acid metabolism, Carbohydrate metabolism, Glucose metabolism

## Abstract

Post-exertional malaise (PEM) is a hallmark symptom of myalgic encephalomyelitis/chronic fatigue syndrome (ME/CFS). We monitored the evolution of 1157 plasma metabolites in 60 ME/CFS (45 female, 15 male) and 45 matched healthy control participants (30 female, 15 male) before and after 2 maximal cardiopulmonary exercise test (CPET) challenges separated by 24 hours, with the intent of provoking PEM in patients. Four time points allowed exploration of the metabolic response to maximal energy-producing capacity and the recovery pattern of participants with ME/CFS compared with the healthy control group. Baseline comparison identified several significantly different metabolites, along with an enriched percentage of yet-to-be identified compounds. Additionally, temporal measures demonstrated an increased metabolic disparity between cohorts, including unknown metabolites. The effects of exertion in the ME/CFS cohort predominantly highlighted lipid-related as well as energy-related pathways and chemical structure clusters, which were disparately affected by the first and second exercise sessions. The 24-hour recovery period was distinct in the ME/CFS cohort, with over a quarter of the identified pathways statistically different from the controls. The pathways that are uniquely different 24 hours after an exercise challenge provide clues to metabolic disruptions that lead to PEM. Numerous altered pathways were observed to depend on glutamate metabolism, a crucial component of the homeostasis of many organs in the body, including the brain.

## Graphical abstract

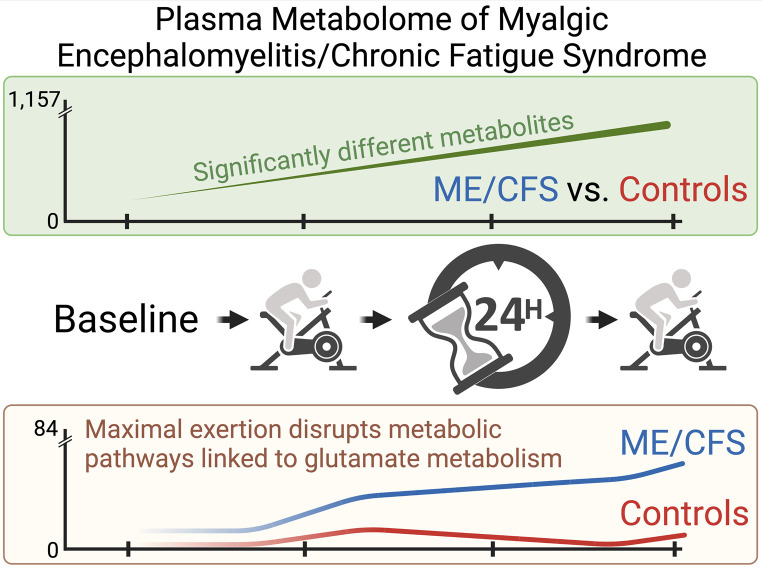

## Introduction

Myalgic encephalomyelitis/chronic fatigue syndrome (ME/CFS) affects and marginalizes a substantial portion of people across the world, often restricting many patients to silence and isolation (1–4). Symptoms of ME/CFS affect many of the 11 organ systems of the body. Although the inciting factor for ME/CFS is not yet known, substantial evidence implicates the disease as a post-viral illness (5). Many symptoms of ME/CFS are shared with long-term sequelae of COVID-19, though individuals experiencing continued effects of SARS-CoV-2 infection also exhibit symptoms not characteristic of ME/CFS (6).

One of the hallmark symptoms preventing ME/CFS sufferers from living normal lives surfaces after physical exertion, as well as after cognitive and/or emotional stimuli (1). This symptom, referred to as post-exertional malaise (PEM), occurs after varying levels of stimulus, proportional to each individual’s limit. The strategy of pacing, adapted to meet individual abilities and limitations, is recognized as a therapeutic option to reduce the severity of exertion-related symptoms (7). PEM typically occurs 12 to 48 hours after physical activity and persists for days or even weeks, confining some patients to bed during recovery (4).

Monitoring plasma metabolomics changes associated with acute physical activity has led to valuable insight into both individual responses to an exertional stressor and to the “molecular choreography” of biological processes in numerous pathways (8). Many studies, including from our group, have probed the metabolome of various body fluids from participants with ME/CFS, at a depth dependent on the technology selected (9–20). However, none have characterized temporal metabolic changes before and after a standardized exertional stressor aimed at inducing PEM. One study of ME/CFS cases found evidence of a shift toward branched-chain amino acids as an energy source during intense exercise (21). A more recent study found an association of PEM with glycolysis, acetylation, and hypermetabolism, based mainly on limited urine and serum metabolomics (19).

The investigation reported here differs from previous studies by combining several important aspects: (i) the large size of the cohort selected, with 105 participants, including 60 participants with ME/CFS (45 women and 15 men) as well as controls screened for sedentary behavior; (ii) the longitudinal blood sampling, surrounding 2 cardiopulmonary exercise tests (CPETs) separated by a 24-hour recovery period designed to provoke PEM; and (iii) the extensive data set produced by Metabolon from its Precision Metabolomics platform composed of 1157 features, including 933 identified and 224 yet-to-be-identified metabolites, spanning 9 superpathways and 108 subpathways.

We identified several significantly differently abundant metabolites between participants with ME/CFS and controls. Pathway and chemical enrichment analyses were the most fruitful aspect of this work, exposing the impact of exertion on the magnitude and the number of differences between participants with ME/CFS and healthy although sedentary individuals. Analysis of plasma metabolites following an initial maximal exercise session and those in plasma collected 24 hours later reveals that participants with ME/CFS exhibit extensive differences with controls during the recovery period. One common denominator between many of these pathways is glutamate metabolism. Our work also confirms divergent patterns between females and males following both exercise and recovery.

## Results

### Provocation of post-exertional malaise.

**Figure 1:**
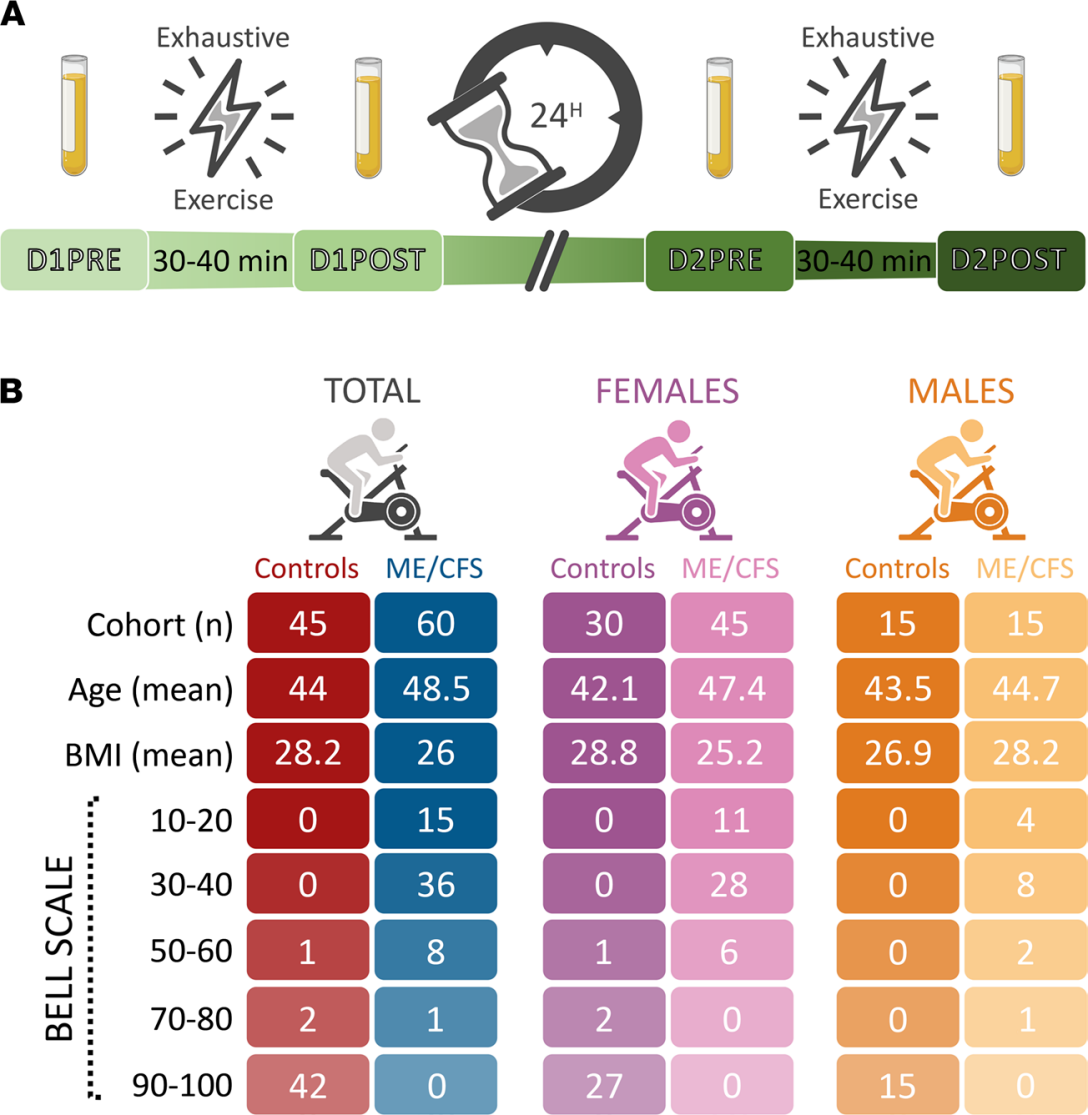
Study design and population statistics. (**A**) Overview of the blood collection timeline surrounding 2 maximal cardiopulmonary exercise tests (CPETs) separated by a 24-hour recovery period. (**B**) Summary of the most representative features of the population. A score of 100 on the Bell scale (2) corresponds to no symptoms at rest, while a score of 0 means continuous symptoms, a bedbound state, and inability to take care of oneself. D1PRE, before exercise on day 1; D1POST, after exercise on day 1; D2PRE, before exercise on day 2; D2POST, after exercise on day 2.

Both participants with ME/CFS and healthy controls performed 2 maximal-effort exercise tests of 6 to 14 minutes on a stationary cycle at a 24-hour interval designed to induce PEM in participants with ME/CFS (Figure 1A). Blood samples were collected 15–20 minutes before exercise on day 1 (D1PRE) and before exercise on day 2 (D2PRE) as well as 15–20 minutes after exercise on day 1 (D1POST) and after exercise on day 2 (D2POST) for a total of 4 time points, as depicted in the timeline of Figure 1A.

### Functional surveys distinguish the ME/CFS and control cohorts.

The 105-participant cohort gathered for this study comprised 60 participants with ME/CFS and 45 healthy, sedentary controls between the ages of 18 and 69, with similar body mass index (BMI). Figure 1B summarizes the most representative features of our cohorts, including the subdivision of women and men, the mean age and BMI, as well as the Bell scale (2) scored by 5 functional levels. The Bell scale score clearly depicts the opposing trend of the condition of participants with ME/CFS versus controls, with a smaller score reflecting the lower functional level of patients compared with a higher score of fully functional controls. Nevertheless, the selected ME/CFS population was capable of traveling to the testing sites and performing 2 CPETs and would therefore be considered to have less severe symptoms compared with patients who are bedbound. However, the scores on the Bell scale and 36-Item Short-Form Health Survey (SF-36) indicate that very few patients could be considered to have mild disease. Comprehensive details about the population characteristics are offered in Supplemental Tables 1 and 2 (supplemental material available online with this article; https://doi.org/10.1172/jci.insight.157621DS1), including illness duration and the results of the SF-36 (22), among other data.

### Global metabolomics panel.

Metabolon assayed the relative levels of 933 known metabolites grouped into its defined 9 superpathways, including “amino acid” (216 compounds), “carbohydrate” (26 compounds), “cofactors and vitamins” (37 compounds), “energy” (11 compounds), “lipid” (293 compounds), “nucleotide” (37 compounds), “partially characterized molecules” (27 compounds), “peptide” (36 compounds), and “xenobiotics” (250 compounds). These 9 superpathways are further split into 108 subpathways detailed in Supplemental Data File 1, which also contains the complete data set Metabolon provided. Additionally, the relative quantities of 224 metabolites of unknown identities (19% of the data set) are provided to allow analysis of currently unidentified metabolites.

### No evidence for general hypometabolism.

We assessed general differences between controls and patients by comparing fold changes (FCs) of means between controls and patients at each time point (FC of 2 reflects a mean twice as high in patients compared with controls for a given metabolite). For the complete cohort, 56% of the metabolites were lower (FC < 1) in patients compared with controls at D1PRE. This percentage marginally dropped throughout our experimental protocol to reach 53% at D2POST. If considering “0.9 < FC < 1.1” as reflecting no change for a given metabolite, about 34% were lower (FC < 0.9) in patients compared with controls, while 29% were higher (FC > 1.1). The distribution of FC for the total cohort and all metabolites is illustrated in Supplemental Figure 1, where we can observe the symmetry of the violin plots around log_2_FC = 0, which is the same as FC = 1, or no change.

In the female cohort, 50% of the metabolites were lower (FC < 1) in patients versus controls at D1PRE. This percentage dropped throughout the remaining time points to reach 43% at D2POST. Excluding metabolites considered constant at 0.9 < FC < 1.1, 30% of metabolites were lower (FC < 0.9) in patients compared with controls for all time points, while 29% were higher (FC > 1.1) at D1PRE, constantly increasing through our timeline to reach 35% at D2POST. Again, the violin plots for the female cohort in Supplemental Figure 1 are symmetric around log_2_FC = 0.

The trend slightly deviated from the above observations in the male cohort. Indeed, 59% of metabolites were lower (FC < 1) in patients compared with controls at D1PRE, 57% at D1POST, 63% at D2PRE, and 61% at D2POST. Moreover, the exclusion of constant metabolites at 0.9 < FC < 1.1 revealed that 44% of remaining metabolites were lower (FC < 0.9) in patients compared with controls, while only 20% were higher (FC > 1.1). The limited size of our male cohort must be considered when interpreting the data on relative levels of metabolites. The violin plots for the male cohort in Supplemental Figure 1 are slightly skewed in favor of negative log_2_FC, meaning more metabolites were lower in male patients compared with male controls.

### High-dimensional clustering groups participants by time points.

**Figure 2:**
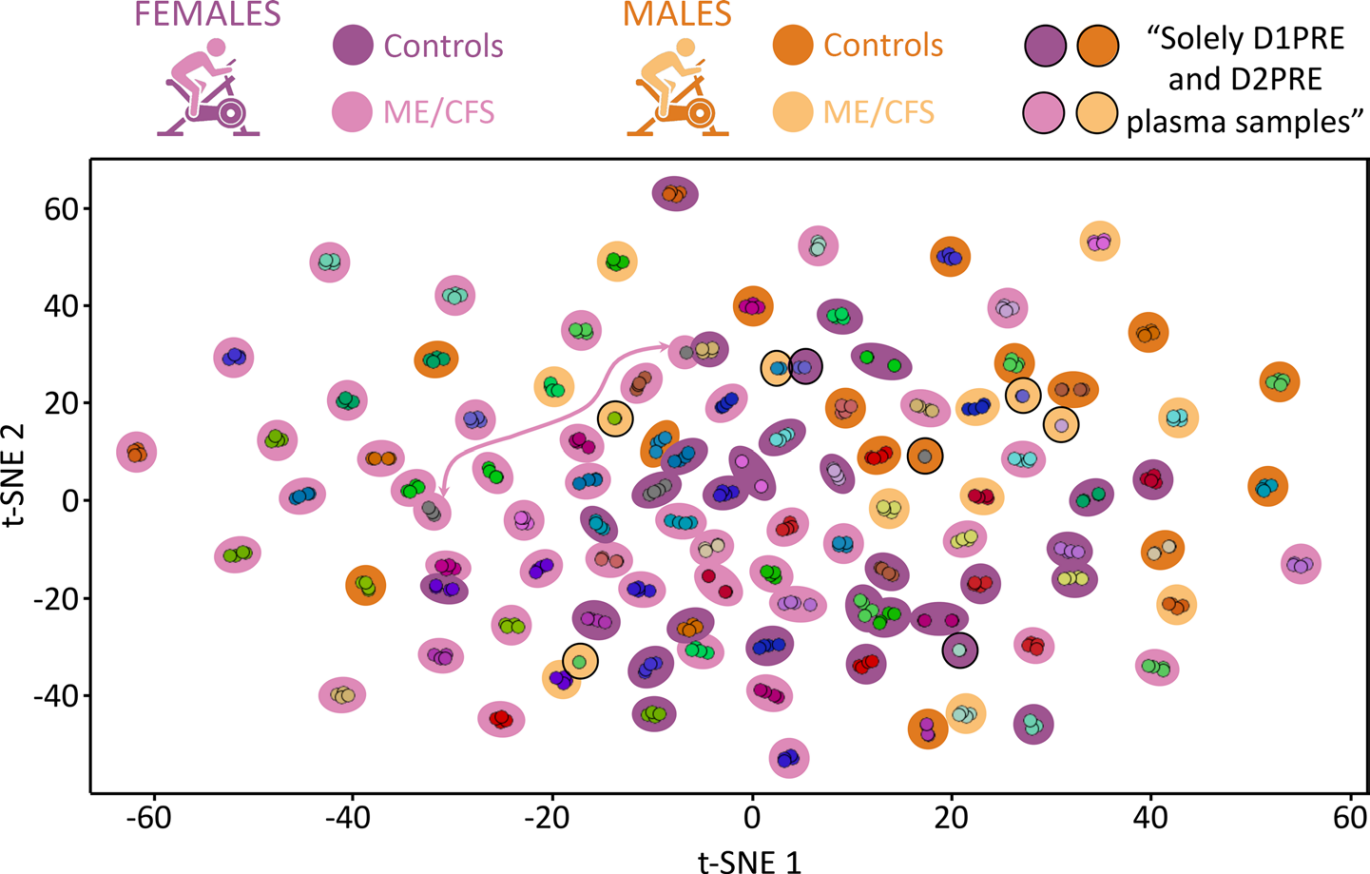
Output from t-SNE using the complete data set. Each dot represents a single plasma sample. Coloring is specific to each participant.

A t-distributed stochastic neighbor embedding (t-SNE) plot shows strong clustering of time points for each participant of our cohort (similarly colored dots in Figure 2), representing each individual’s plasma metabolic environment. However, superimposing sample phenotypes and sexes did not reveal any clustering. We also never observed any clustering by sex or disease status after comparable iterations of clustering attempts were performed using sub–data sets created through various criteria using the comparable principal component analysis (PCA) method (not shown).

Both the t-SNE and PCA methods aim to reduce the complexity of a data set and retain the features that best explain variability. The strong but sole clustering of time points by participant reflected the robust individuality of the plasma metabolic environment, even as strong physical exertion occurred. This is identical to the clustering Contrepois et al. described in their multidisciplinary molecular choreography work (8).

### The number of metabolites statistically different between cohorts increases after exercise in a sex-specific manner.

**Figure 3:**
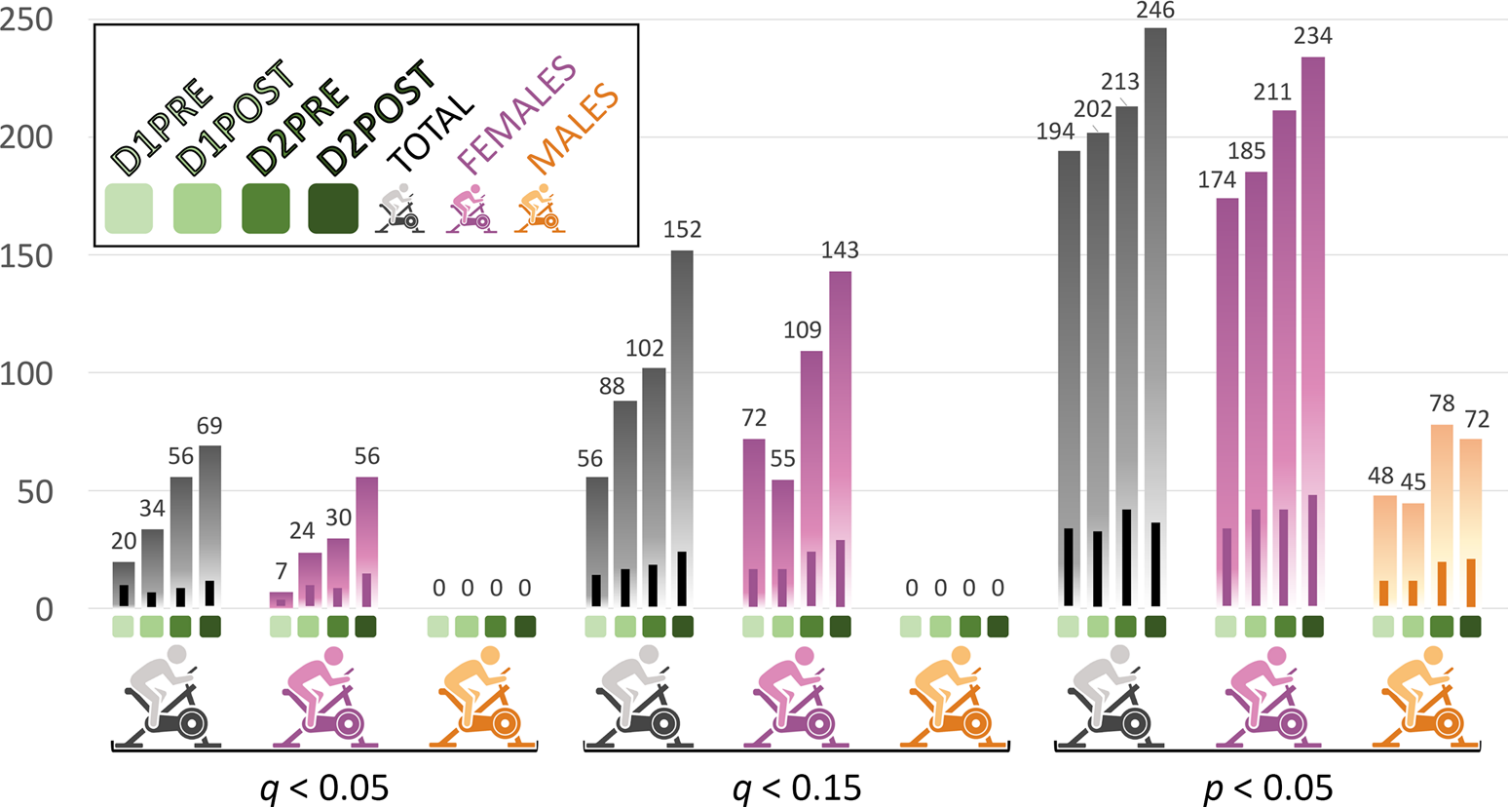
Bar graph of the number of significantly different metabolites between controls and participants with ME/CFS based on Wilcoxon’s testing, at each time point for the 3 cohorts (total, women, and men), and for 3 statistical cutoffs (*q* < 0.05, *q* < 0.15, and *P* < 0.05). The bars within the histogram bars represent the number of unknown metabolites for each cohort and each time point.

The summary of nonparametric Wilcoxon’s rank-sum tests at each time point is displayed in Figure 3 for each cohort at 3 statistical cutoffs: *q* < 0.05, *q* < 0.15, and *P* < 0.05. Details concerning our choice of multiple statistical cutoffs are in the Methods section, while the complete output is available in Supplemental Table 4.

The total cohort (Figure 3, gray bars) depicts an increasing number of metabolites significantly different between controls and patients as the study proceeded, for all 3 of the statistical cutoff values. This sequence reflected the escalating differences between cohorts during progression through our experimental protocol. Similarly, for the female cohort (Figure 3, purple bars) at *q* < 0.05, there were 8 times more significantly different metabolites between D1PRE and D2POST. Although a constant but more moderate increase also existed at *P* < 0.05, a slight decrease of significantly different metabolites at D1POST compared with D1PRE was observed for *q* < 0.15.

On the other hand, statistically significant metabolic differences were identified only at *P* < 0.05 in the male cohort (Figure 3, orange bars). A notable difference compared with the female cohort (Figure 3, purple bars) was the doubling of significantly different metabolites during the recovery period, while the exercise neither on day 1 nor the exercise on day 2 amplified the difference in plasma metabolites (the number of significantly different metabolites was actually a little lower after each set of exercise).

### Significantly different metabolites are predominantly lower in patients.

**Figure 4:**
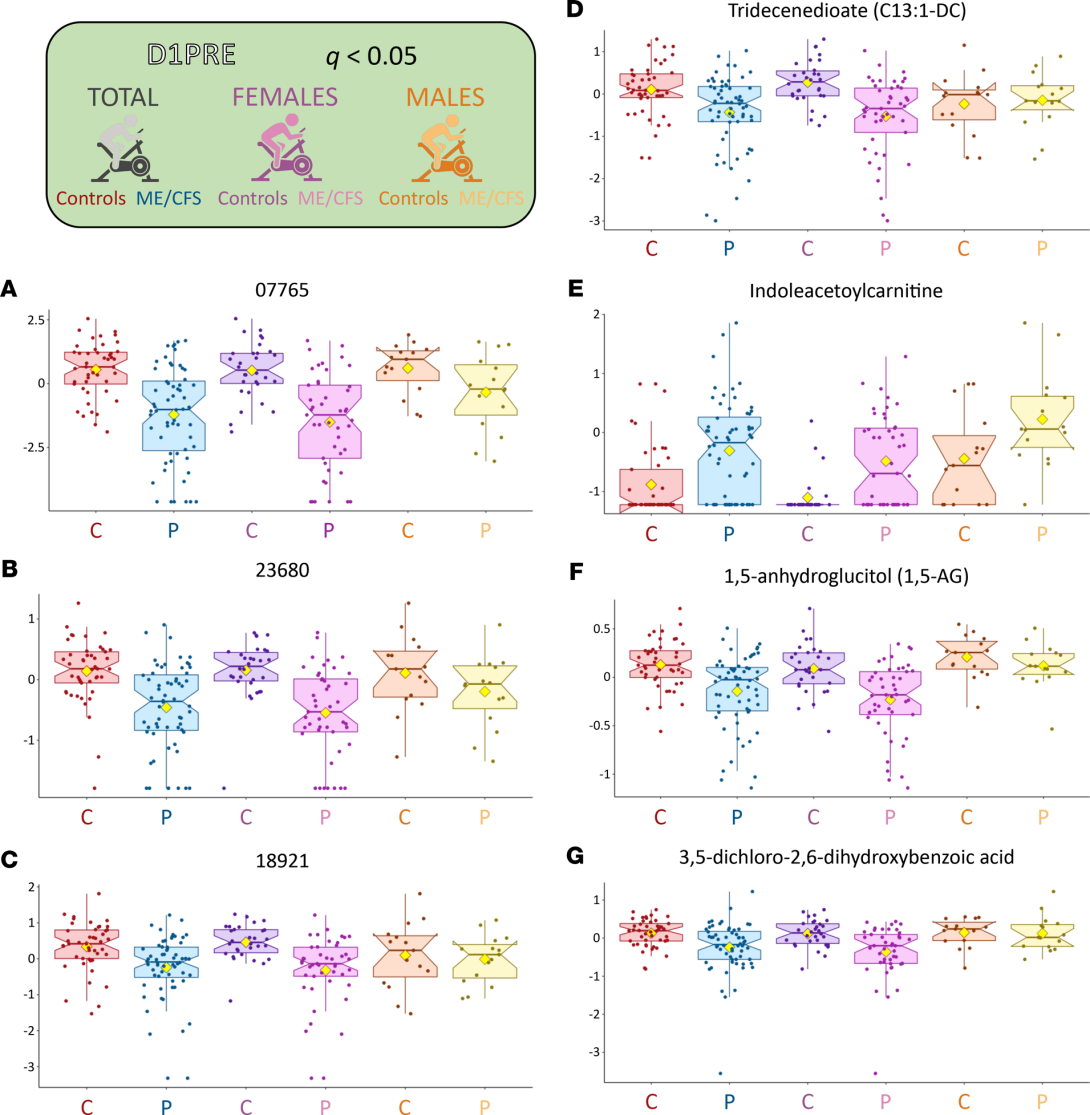
Box plots of metabolites significantly different between female controls and patients at D1PRE for *q* < 0. 05. The yellow diamond represents the mean of the logged values. Plots depict the minimum and maximum values (whiskers) and interquartile range (length of filled shape). (**A**–**C**) Unknowns. (**D**) Tridecenedioate. (**E**) Indoleacetoylcarnitine. (**F**) 1,5-anhydroglucitol (1,5-AG). (**G**) 3,5-dichloro-2,6-dihydroxybenzoic acid.

The metabolites with the highest statistical differences (Figure 3) were generally lower in individuals with ME/CFS. For instance, for *q* < 0.05 at D1PRE, 86% of the 7 metabolites were lower in female participants while 5 (71%) were also lower in male participants (Figure 4). At *P* < 0.05 and D1PRE, 75% of metabolites were lower in female participants while 67% were lower at D2POST. Further details can be computed from Supplemental Table 4 for each cohort, each time point, and at any provided statistical cutoff.

### At baseline, 7 metabolites out of 1157 (0.6%) are highly significantly different in women.

The data distribution of the 7 metabolites significantly different out of the 1157 (0.6%) after multiple-testing correction (*q* < 0.05) is shown in Figure 4 for all 3 cohorts (total, women, and men) at D1PRE.

Metabolites of unknown identity were the 3 most significantly different metabolites (Figure 4, A–C). Although 07765 and 23680 had similar patterns between the 3 cohorts, male patients and controls did not differ for 18921. Out of the next 4, tridecenedioate (C13:1-DC) (Figure 4D) is classified as a “fatty acid dicarboxylate” (part of lipid); indoleacetoylcarnitine (Figure 4E) as an “amino acid” part of “tryptophan metabolism”; 1,5-anhydroglucitol (1,5-AG) (Figure 4F) as a “carbohydrate” part of “glycolysis, gluconeogenesis, and pyruvate metabolism”; and 3,5-dichloro-2,6-dihydroxybenzoic acid (Figure 4G) as a “chemical xenobiotic.”

The only significant baseline metabolite with an associated Human Metabolome Database (HMDB) number (HMDB0002712) is 1,5-AG. According to the curated database (https://hmdb.ca), 1,5-AG is validated as a blood marker of glycemic control, because it is metabolically inert and it competes with glucose reabsorption in the kidney. It can be used as a proxy to monitor long-term fluctuations of glucose in the blood.

### Unknown metabolites are enriched among significantly different metabolites.

The number of metabolites of unknown identity (19% of our data set) increased with statistical significance. The bars within the histograms in Figure 3 represent the number of unknown metabolites while percentages are provided in Supplemental Table 3.

As mentioned above for the female cohort in Figures 3 and 4, 3 out of 7 metabolites from D1PRE at *q* < 0.05 were of unknown identity (43%). At D1POST, it was 9 out of 24 (38%) and for D2PRE and D2POST around 26%. At the higher threshold of *q* < 0.15, the percentages dropped to 22%, 29%, 21%, and 20%, respectively, out of the numbers graphed in Figure 3. At *P* < 0.05, the percentages averaged around 19% (Supplemental Table 3).

Concerning the total cohort, apart from D1PRE at *q* < 0.05 where 9 out of 20 metabolites were unknowns (45%), the percentages for the other time points and statistical thresholds hovered under 19% (from 14 to 18%, Supplemental Table 3).

However, for the only threshold with significantly different metabolites in male participants (*P* < 0.05 in Figure 3), the trend was distinctive, with percentages of unknowns slowly increasing from 23% for D1PRE, to 24% for both D1POST and D2PRE and 28% for D2POST (Supplemental Table 3).

### Overlap analysis reveals sex-dependent patterns.

**Figure 5:**
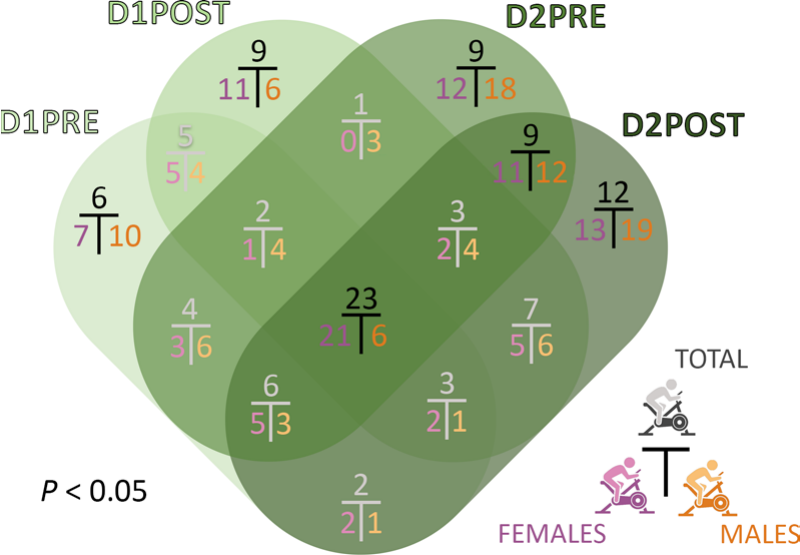
Venn diagram of the overlap analysis performed for each cohort between the 4 time points for metabolites statistically different between participants with ME/CFS and controls at *P* < 0.05. The numbers shown are percentages to respect the proportionality between the smaller number of metabolites for the male cohort and both the female and total cohort, which have 3 times as many. Darkened percentages are the ones mentioned in the text.

One important detail missing from Figure 3 is the identity overlap of significantly different metabolites between each time point within each cohort. We investigated this overlap at the statistical threshold of *P* < 0.05 to include the male cohort, which at *q* < 0.05 or *q* < 0.15 did not include any metabolites (Figure 3). The Venn diagram in Figure 5 depicts the percentages of unique metabolites across the 4 time points for each cohort (383 for total, 385 for women, and 142 for men). Percentages are used to take into account the smaller number of metabolites in the male cohort (Figure 3). Of prime interest to us are the darkened percentages, which represent 75% and 70% of metabolite distinctions in the female and the male cohort, respectively.

The first observation is that the male cohort pattern for the darkened percentages of the Venn diagram differed from the total and female cohort percentages. The exception is for the D2PRE/D2POST intersection, where the percentage was approximately 10% for all cohorts. Those metabolites include changes occurring specifically 24 hours after the initial maximal CPET, independently of the second maximal CPET.

The intersection of all time points at the center of the Venn diagram is at 21% for women and 6% for men. In other words, a fifth of the metabolites are independent from exercise stress in the female cohort, while that proportion is only one-twentieth in the male cohort.

Distinctive patterns between women and men are displayed in the outer parts of the Venn diagram, specific to each time point. Although 7% of the metabolites different in the female cohort were specific to D1PRE, the percentage increased to 11% at D1POST, 12% at D2PRE, and 13% at D2POST. This reflects a gradual transformation of the plasma metabolome as the effect of the exercise stress progressed in women. On the contrary, the male percentages were 10% for D1PRE and 6% for D1POST before jumping to 18% after a 24-hour recovery period (D2PRE) and to 19% at D2POST. In summary, 49% of the male plasma metabolome transformations occur during the 24-hour recovery period and during the exercise on the second day.

### Pathway analysis at baseline reveals the pathways ME/CFS influenced.

**Figure 6:**
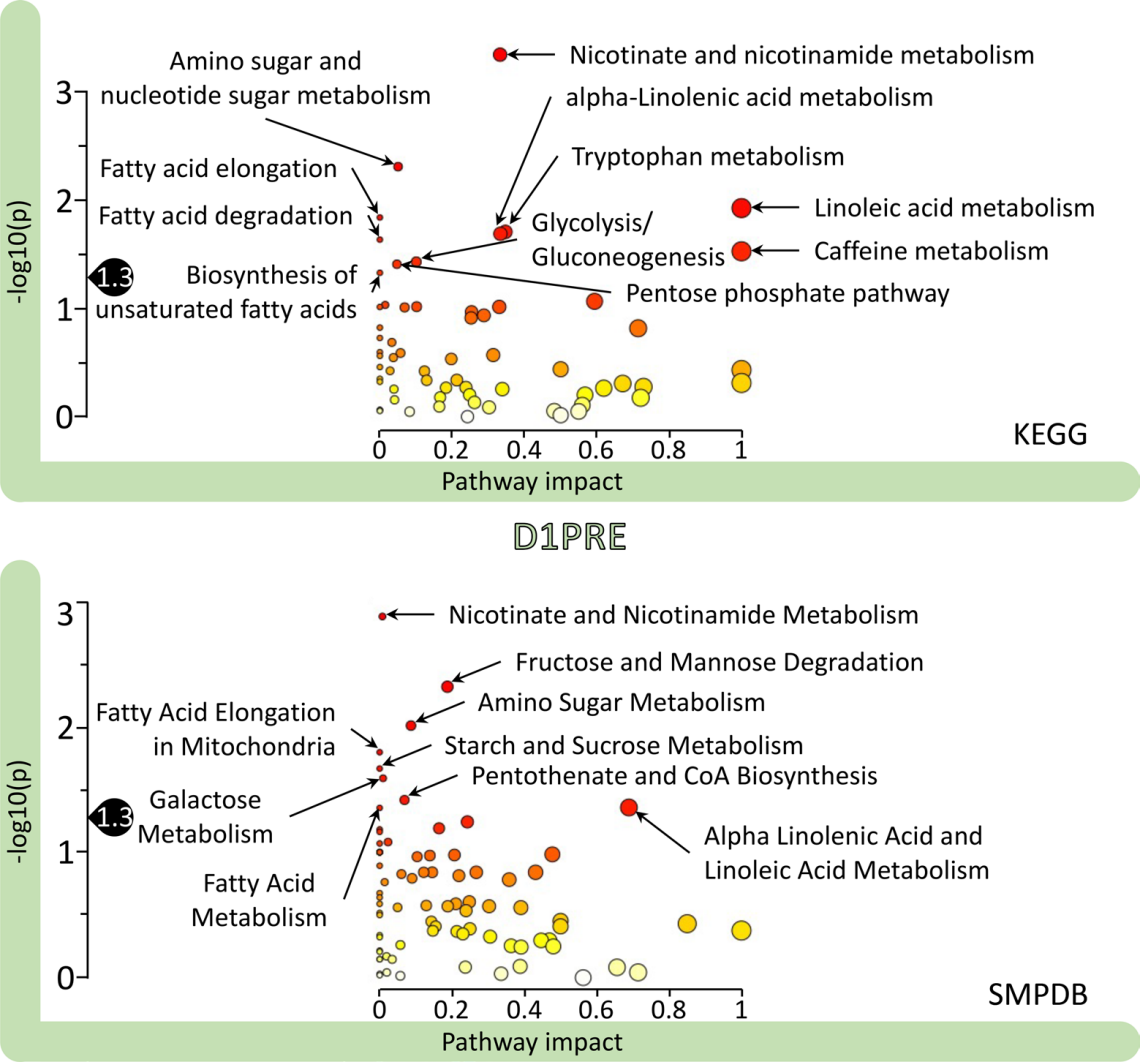
Pathway analysis results using MetaboAnalyst for the female D1PRE time point. The pathway impact on the *y* axis from 0 (low impact) to 1 (strong impact) represents the values from the pathway topology analysis. Each circle denotes a pathway, and the fill color represents the significance of disturbances in that pathway from white (low significance) to red (higher significance). The black drop with 1.3 indicates the threshold of significance at *P* < 0.05.

Because an HMDB number is necessary for classification purposes in this analysis, only 618 out of the 933 known metabolites could be included. We mapped those metabolite values using the Pathway Analysis module from MetaboAnalyst against 2 libraries: Kyoto Encyclopedia of Genes and Genomes (KEGG) and Small Molecule Pathway Database (SMPDB). Each panel of Figure 6 compares controls to participants with ME/CFS and is an independent output for either of the KEGG and SMPDB libraries containing 64 or 84 pathways, respectively. The *x* axis sorts the pathways by pathway impact while the *y* axis represents *P* values. Additional details including unlabeled pathways’ names in Figure 6 are available in Supplemental Table 5.

Although the KEGG and SMPDB libraries label some pathways differently, there are many commonalities when comparing each panel in Figure 6. First, the pathway labeled “nicotinate and nicotinamide metabolism” is common to both and, in both cases, significantly different between controls and patients (KEGG *P* = 0.0006, *q* = 0.04; and SMPDB *P* = 0.001, *q* = 0.1). Nicotinate and nicotinamide, more widely known as vitamin B_3_, are precursors of NAD+ and NADP+, which are essential for redox reactions and carrying electrons between reactions.

We grouped some of the other labeled pathways (all with *P* < 0.05 or –log_10_[0.05] = 1.3; above the black “drops” on the *y* axis in Figure 6) under a category related to energy and sugars. They include “amino sugar and nucleotide sugar metabolism,” “glycolysis/gluconeogenesis,” and “pentose phosphate pathway” in the KEGG panel. In the SMPDB panel, they are “fructose and mannose degradation,” “amino sugar metabolism,” “starch and sucrose metabolism,” and “galactose metabolism.” The KEGG “pentose phosphate pathway” is also an important producer of cytoplasmic NADPH.

A second category that is evident in Figure 6 encompasses lipids, including “alpha-linolenic acid metabolism,” “linoleic acid metabolism,” “fatty acid elongation,” “fatty acid degradation,” and “biosynthesis of unsaturated fatty acids” in the KEGG panel. The SMPDB panel contains similar pathways, such as “fatty acid elongation in mitochondria,” “fatty acid metabolism,” “pentothenate and CoA biosynthesis,” as well as “alpha linoleic acid and linolenic acid metabolism.” Pentothenate, or vitamin B_5_, is a precursor of CoA, an essential molecule in fatty acid metabolism.

The outputs from the analogous analysis for each of the other 4 time points are gathered in Supplemental Figure 2 using the KEGG library. This figure exhibits the changes occurring throughout our experimental protocol. Although some pathways were not affected, others were affected by either one or both exercises; still others were affected during the recovery period. Independent of those fluctuations was the increase in significant differences between controls and participants with ME/CFS as they underwent the experimental protocol. Indeed, an increased number of pathways reached significance (*P* < 0.05 or –log_10_[0.05] = 1.3), with 11 pathways at D1PRE, 19 at D1POST, 17 at D2PRE, and 28 at D2POST. This observation is complementary to the conclusion illustrated by Figure 3.

### Exercise influences the ME/CFS female cohort differently on day 1 versus day 2.

**Figure 7:**
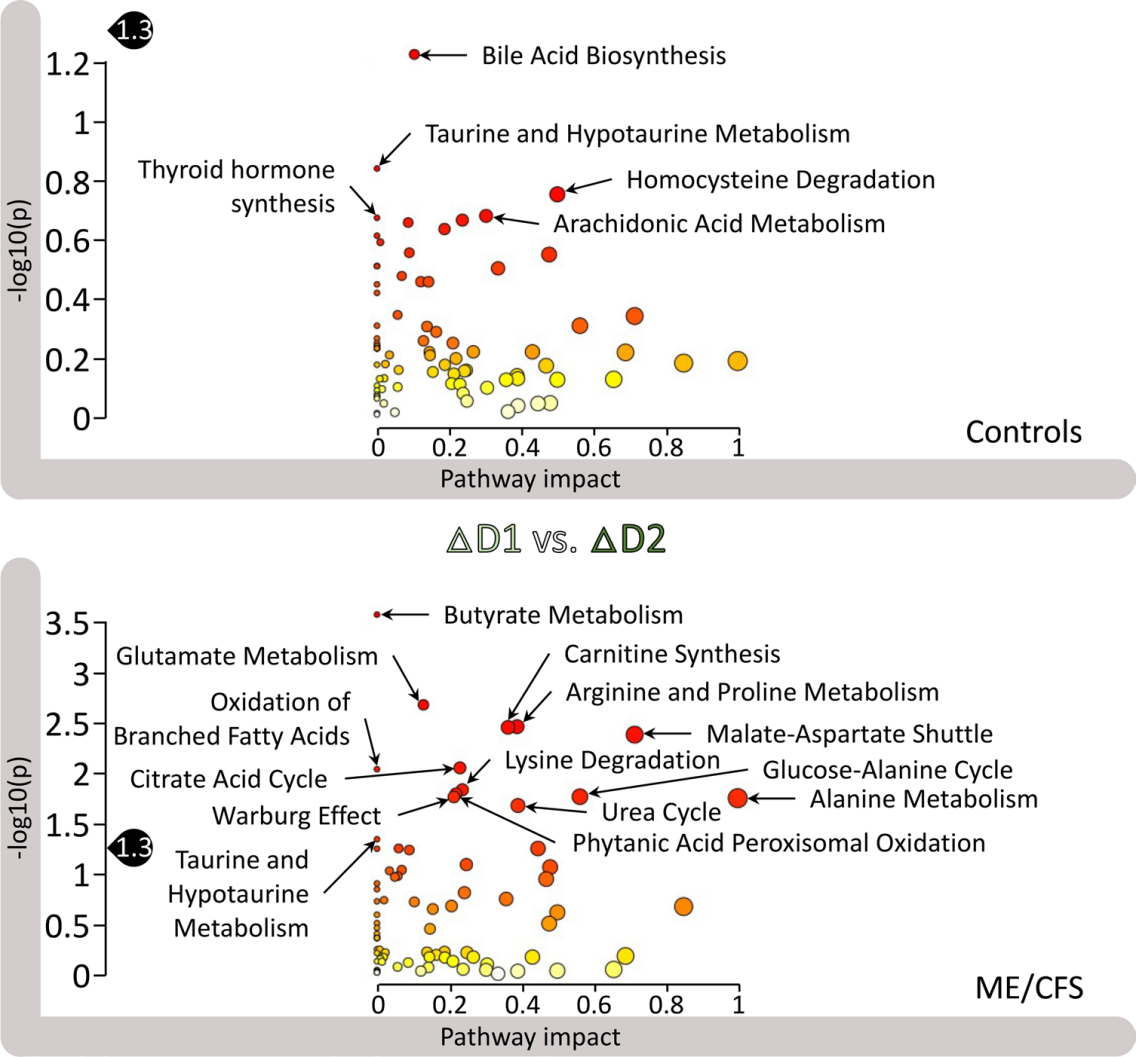
Pathway analysis results from MetaboAnalyst of ΔD1 versus ΔD2 for female controls and participants with ME/CFS (ΔD1 and ΔD2 result from the subtraction for each participant of the D1PRE or D2PRE values from the D1POST and D2POST values for each metabolite). The pathway impact on the *y* axis from 0 (low impact) to 1 (strong impact) represents the values from the pathway topology analysis. Each circle denotes a pathway, and the fill color represents the significance of disturbances in that pathway from white (low significance) to red (higher significance). The black drop with 1.3 indicates the threshold of significance at *P* < 0.05.

We used the same MetaboAnalyst module to investigate a particular data set against the SMPDB library. For this analysis we examined the FC differences between the effect of exercise on day 1, ΔD1, and the effect of exercise on day 2, ΔD2, for both the controls (top panel of Figure 7) and participants with ME/CFS (bottom panel of Figure 7), in the female cohort.

The most striking observation is the lack of significant differences in the controls (no *P* < 0.05). This suggests that the consequences of exercise on day 1 and day 2 are similar for the control cohort. We labeled the top pathways for comparison with the ME/CFS panel, which features 14 pathways with significant FC differences (*P* < 0.05) between the 2 maximal CPETs, including “butyrate metabolism” (*q* = 0.02). The next 6 pathways have a *q* < 0.1, and the subsequent 6 pathways have a *q* < 0.15 (all 14 pathways are labeled in Figure 7). In contrast to the controls, the influence of day 1 exercise and day 2 exercise on the ME/CFS plasma metabolome is markedly distinct.

Glutamate is a common factor for many of the top pathways according to the curated SMPDB, including “butyrate metabolism,” where many of the molecules intestinal fermentation produces can be used for glutamate synthesis. Glutamate is a precursor for arginine and proline and is linked to lysine and alanine metabolism. Glutamate is also a precursor to many nucleic acids and proteins in addition to its role in the central nervous system, where it is an excitatory neurotransmitter and plays a role in neuronal plasticity. Dysfunctional glutamate metabolism can cause many disorders affecting vision, hemolysis (red blood cell destruction), disrupted nitrogen metabolism, or the accumulation of toxic metabolites in the brain, to name a few from the curated SMPDB description.

The pathways “butyrate metabolism,” “carnitine synthesis,” “oxidation of branched-chain fatty acids,” and “phytanic acid peroxisomal oxidation” are all related to lipid metabolism and ultimately to energy production, once products that are generated, such as acetyl-CoA, enter the TCA cycle.

Finally, 2 systems essential to energy production were affected differently between exercise on day 1 and exercise on day 2 in participants with ME/CFS. One was the “malate-aspartate shuttle,” which is essential for the migration of electrons through the impermeable mitochondrial membrane with the contribution of malate and NADH. The other was the “glucose-alanine cycle,” which is involved in muscle protein degradation, generating glutamate and then alanine, the latter of which, after being transported to the liver, is used in gluconeogenesis to produce glucose that can then be transported back to muscles and provide energy, according to the curated SMPDB description.

The same analysis performed on the limited male cohort did not yield any significant results for either patients or controls (not shown).

### The recovery process is highly disrupted in participants with ME/CFS.

**Figure 8:**
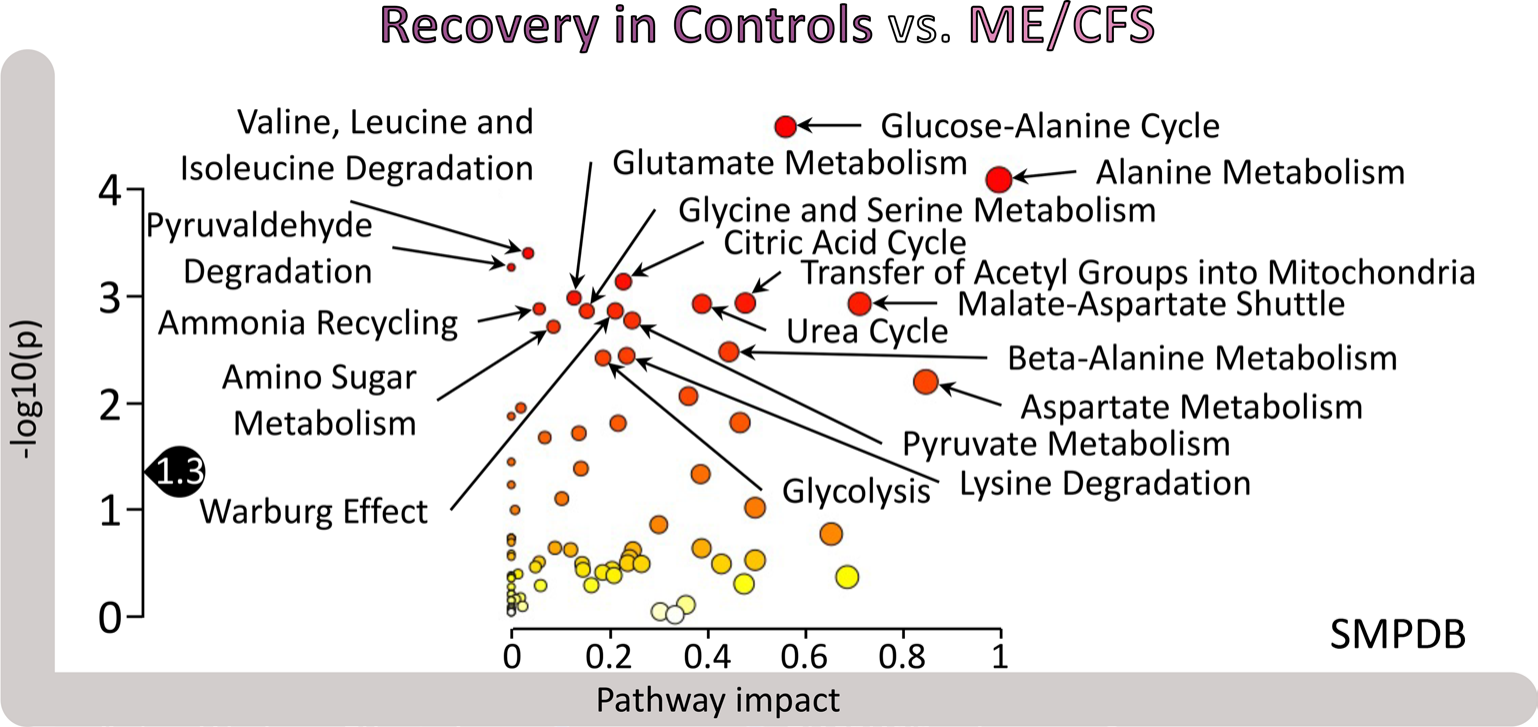
Pathway analysis results from MetaboAnalyst of the recovery period differences between female controls and participants with ME/CFS (the recovery values result from the subtraction for each participant of the D2PRE values from the D1POST values for each metabolite). The pathway impact on the *y* axis from 0 (low impact) to 1 (strong impact) represents the values from the pathway topology analysis. Each circle denotes a pathway, and the fill color represents the significance of disturbances in that pathway from white (low significance) to red (higher significance). The black drop with 1.3 indicates the threshold of significance at *P* < 0.05.

We tested the FC differences between the recovery in controls and the recovery in participants with ME/CFS against the SMPDB library. Out of the 28 significantly different pathways at *P* < 0.05, 20 were also significant at *q* < 0.05 and all at *q* < 0.15. The top 18 are labeled in Figure 8, with many familiar actors in energy metabolism, including “glucose-alanine cycle,” “citric acid cycle,” “malate-aspartate shuttle,” “pyruvate metabolism,” “amino sugar metabolism,” as well as “glycolysis.”

Although pathways related to lipids were not enriched in this output, another few notable ones were “urea cycle” and “ammonia recycling,” which are related to nitrogen metabolism.

The same analysis for the limited male cohort yielded 7 significantly different pathways (*P* < 0.05), including “phosphatidylethanolamine biosynthesis” and “phosphatidylcholine biosynthesis,” both lipid related, as well as “galactose metabolism,” “fructose and mannose degradation,” and “starch and sucrose metabolism” (output not shown).

The facts that the 24-hour recovery data gave rise to the only output that generated significant results in men, and that there were many significantly differences for the female cohort, clearly illustrate the fundamentally affected physiology in exercise recovery in our ME/CFS cohort compared with our healthy, sedentary cohort.

### A chemical ontology analysis reveals the baseline disparities ME/CFS causes.

**Figure 9:**
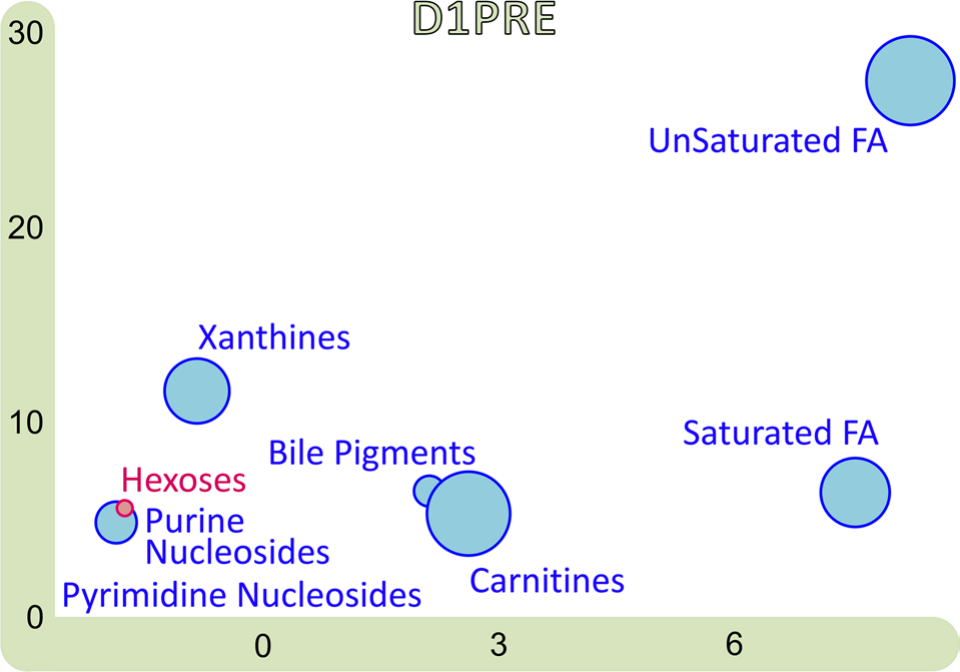
ChemRICH output of D1PRE for the female cohort. Only clusters enriched at *P* < 0.05 are shown. The *x* axis is the cluster order on the similarity tree. The *y* axis is the –log(*P* value), with the most significantly altered clusters at the top. The color scale represents the portion of metabolites with a ratio of patients/controls either decreased (in blue) or increased (in red).

For this inquiry, we used the statistical enrichment approach based on chemical similarity provided by the online tool ChemRICH (http://chemrich.fiehnlab.ucdavis.edu). Because of the required input fields, only 672 out of the 933 identified metabolites could be included in this analysis. The output from the female data set revealed 8 clusters enriched at D1PRE (Figure 9). Seven of them contained metabolites that were decreased in patients compared with controls (blue clusters), while the “hexoses” cluster was the only one containing metabolites with increased amounts in patients’ plasma compared with controls, pinpointing a dysfunction in carbohydrate metabolism. “Purine nucleosides,” “pyrimidine nucleosides,” and “xanthines” are all clusters related to nucleic acids, while metabolites in the “bile pigments” cluster are formed during heme degradation. The remaining clusters, “carnitines,” “unsaturated fatty acids,” and “saturated fatty acids,” demonstrated strong disruptions in lipid metabolism (large circles reflect more disrupted metabolites belonging to that cluster).

A matching ChemRICH analysis with the male data set resulted in very few clusters, namely “sugar alcohols” and “glycerophospholipids” for D1PRE. The difference in output between the male and the female cohort is again most likely due to the limited size of the male cohort, hindering the statistical power of this tool.

### A chemical ontology analysis reveals the numerous changes occurring following exercise.

ChemRICH was applied to the remaining 3 time points (Supplemental Figures 3–5). Out of the 8 enriched clusters at D1PRE, “xanthines,” “carnitines,” and “unsaturated fatty acids” were enriched throughout the 4 time points. It is important to note that the color of the “carnitines” cluster is purple at D2POST after being blue at all the other 3 time points, reflecting a shift in some metabolites from that cluster becoming higher in participants with ME/CFS than controls.

At D1POST (Supplemental Figure 3), 9 metabolic clusters were enriched, and 3 of them were increased in ME/CFS cases, namely “hexoses,” “oligosaccharides,” and “glycerophospholipids.”

At D2PRE and D2POST (Supplemental Figures 4 and 5), the number of enriched metabolic clusters was considerably higher, with 15 and 17, respectively, and in both cases 53% were higher in participants with ME/CFS than controls. Four clusters were specific to those time points, “pyruvates,” “dicarboxylic acids,” “deoxycholic acids,” and “cholic acids.” The last 3 are bile acids, which are produced to help the digestion of fats.

The same analysis with the male cohort resulted in no enriched cluster at D1POST; “oligosaccharides,” “trimethyl ammonium compounds,” and “pyruvates” at D2PRE; and “adenine nucleotides” at D2POST (output not shown).

### Chemical ontology analysis throughout our temporal sampling exposes disparities between participants with ME/CFS and healthy controls.

**Figure 10:**
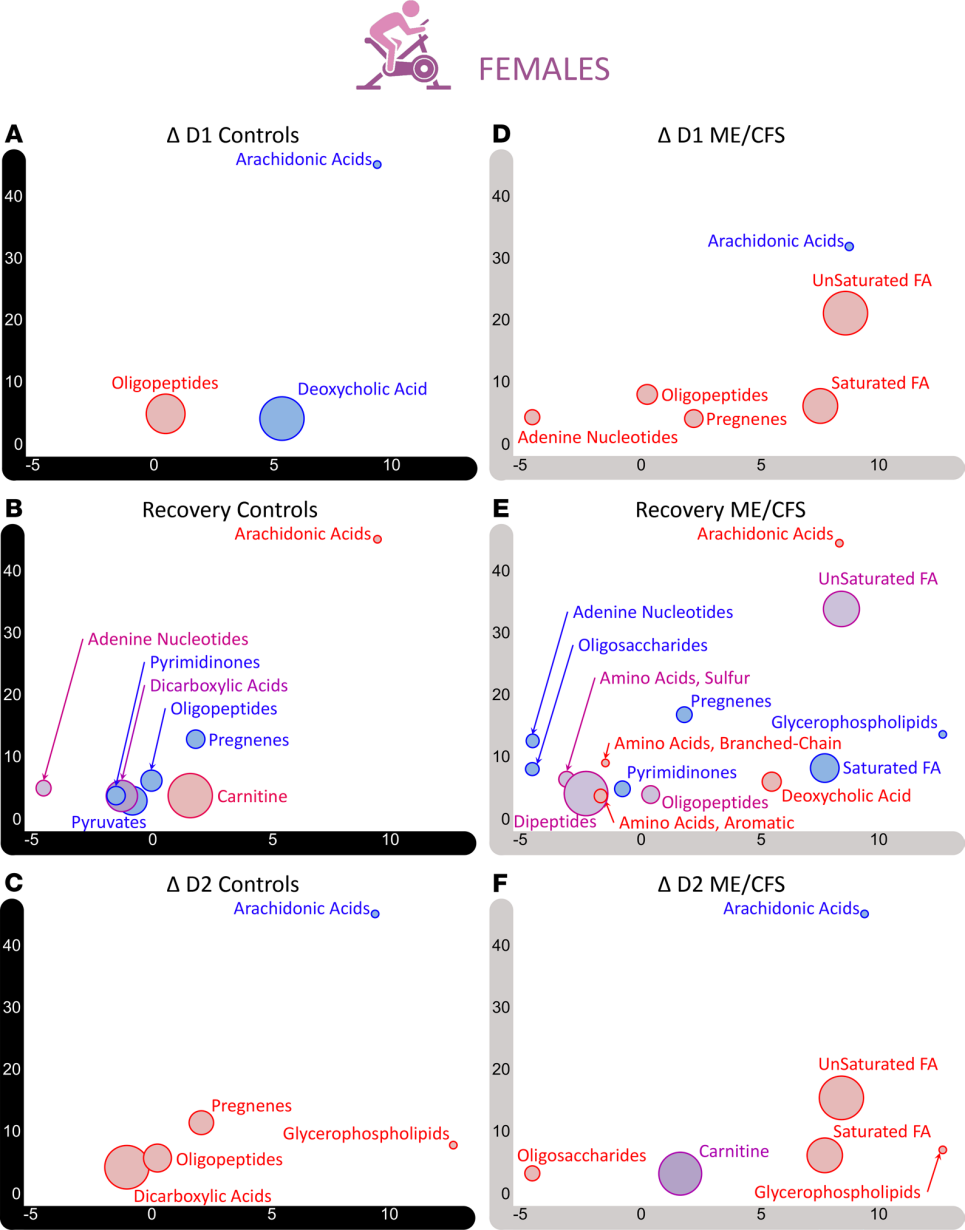
ChemRICH output of ΔD1, the 24-hour recovery period, and ΔD2 for female controls and participants with ME/CFS (ΔD1 and ΔD2 result from the subtraction for each participant of the D1PRE or D2PRE values from the D1POST and D2POST values for each metabolite, while the recovery values result from the subtraction for each participant of the D2PRE values from the D1POST values for each metabolite). (**A**) ΔD1 controls. (**B**) Recovery controls. (**C**) ΔD2 controls. (**D**) ΔD1 ME/CFS. (**E**) Recovery ME/CFS. (**F**) ΔD2 ME/CFS. Only clusters enriched at *P* < 0.05 are shown. The *x* axis is the cluster order on the similarity tree. The *y* axis is the –log(*P* value), with the most significantly altered clusters at the top. The color scale represents the portion of metabolites with a ratio of patients/controls either decreased (in blue) or increased (in red) or an equal number of metabolites both increased and decreased (in purple).

We investigated the impact of exercise on day 1 (ΔD1), during the 24-hour recovery period, as well as the impact of exercise on day 2 (ΔD2) for female controls and participants with ME/CFS using the ChemRICH tool (Figure 10). The only common pattern between all panels of Figure 10 is the “arachidonic acids” cluster, which contains metabolites that decreased because of both exercise bouts but then increased during the recovery period. The 3 metabolites included in this cluster are oleoylcholine, arachidonoylcholine, and linoleoylcholine, all classified as “acyl cholines” (part of lipid) by Metabolon. The pattern of this cluster is a classic example of changes driven solely by our experimental protocol.

The “oligopeptides” cluster behaved in the opposite pattern for the control cohort (Figure 10, A–C). Although it was also an enriched cluster in Figure 10D, its pattern differed during recovery of the ME/CFS cohort (Figure 10E), with some metabolites not recovering like others and the cluster being absent following exercise on day 2 (Figure 10F).

Inversely, the “unsaturated fatty acids” and “saturated fatty acids” clusters were not significantly enriched in the control cohort while they displayed a similar pattern to the “oligopeptides” cluster in controls. Indeed, their metabolites increased because of exercise on both days and recovery during the 24-hour period between them, even if only for certain metabolites for the “unsaturated fatty acids” cluster (purple coloring in Figure 10E).

These are only a few examples of the abundant disparities between controls and participants with ME/CFS depicted in Figure 10. There were 8 clusters significantly enriched during the recovery period in controls versus 14 for the patients, with 5 in common strictly by name (“arachidonic acids,” “adenine nucleotides,” “pyrimidinones,” “oligopeptides,” and “pregnenes”), and only 3 behaving similarly, based on color (“arachidonic acids,” “pyrimidinones,” and “pregnenes”).

The identical analysis on the male cohort delivered contrasting outputs (Supplemental Figure 6), where most of the changes were detected in the control cohort, particularly during the 24-hour recovery period (Supplemental Figure 5B). “Arachidonic acids” is a familiar cluster that behaved identically to the female cohort throughout the 6 panels. In general, clusters from the male control cohort contained metabolites that increased because of exercise on day 1 (Supplemental Figure 5A), then recovered during the recovery period (Supplemental Figure 6B), while few significant changes occurred because of exercise on day 2 (Supplemental Figure 6C). The scarcity of significant clusters in the ME/CFS panels (Supplemental Figure 6, D–F) could reflect a sex-related difference but is most likely the result of a mathematical artifact because of the lack of samples available for analysis for the post-exercise time points (see Methods).

Combining both cohorts to perform the same analysis produced a complex output available as Supplemental Figure 7. One remarkable feature is the strong similarity between controls and participants with ME/CFS for ΔD2, in both cluster identity and behaviors (Supplemental Figure 7, C and F). In contrary, the 24-hour recovery panels show very few commonalities apart from the clusters mentioned above for both the female and the male cohort analysis (Supplemental Figure 7, B and E). Surprisingly, the ΔD1 panels diverge only by a few clusters, including the presence of “carnitine” in controls and “unsaturated fatty acids” in participants with ME/CFS (Supplemental Figure 7, A and D).

### A linear model analysis identifies metabolites with the most distinct profiles.

We utilized an R script designed to test the significance of mean differences (*P* and *q* values) within and between cohorts for all time points. The comparisons include (i) controls versus patients at each time point, (ii) time point to time point for each cohort, as well as (iii) time point to time point for controls versus patients, for a total of 22 combinations/columns. The extensive results for the female cohort are provided as Supplemental Table 6.

**Figure 11:**
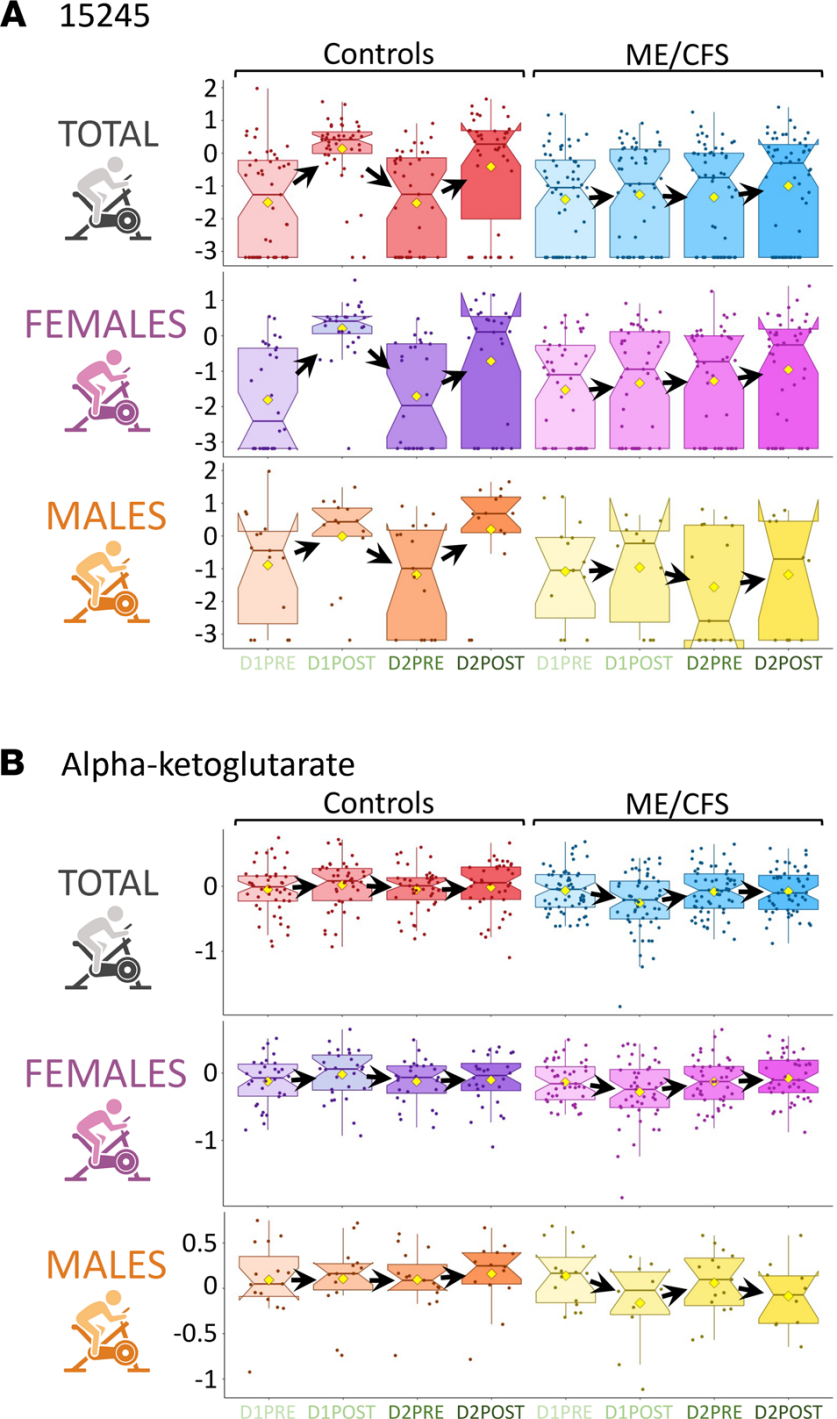
Box plot distribution of logged values for the top 2 metabolites with the most significant differences for controls versus participants with ME/CFS between D1PRE and D1POST. (**A**) 15245. (**B**) Alpha-ketoglutarate. The yellow diamond represents the mean of the logged values. Plots depict the minimum and maximum values (whiskers) and interquartile range (length of filled shape). The black arrows follow the changes of the means of the logged values.

For D1PRE to D1POST changes of controls versus patients, with *q* < 0.15, 11 metabolites were of interest (the prior top 15, mostly drugs, were statistical artifacts). Out of the 11 metabolites, the highest significance was for a metabolite of unknown identity, 15245 (Figure 11A). The second most highly significant is an energy metabolite from the TCA cycle, alpha-ketoglutarate (Figure 11B). Additionally, 2 of them, pyruvate and lactate, are carbohydrates classified as part of “glycolysis, gluconeogenesis, and pyruvate metabolism.” Six of them are amino acids, with 3 being conjugates of 1-carboxyethyl amino acids (leucine, tyrosine, and valine).

Concerning 15245 (Figure 11A), the data distribution between D1PRE and D1POST is clearly different between controls and patients, with a higher mean and median after exercise in controls compared with more constant values for patients. Although similar, this disparity is even more pronounced for women than men. The pattern is analogous from D2PRE to D2POST but not as distinct in female participants. Supplemental Table 6 denotes such slight variations by showing both the *P* and *q* values.

Alpha-ketoglutarate (Figure 11B) displays an opposite pattern between controls, increasing due to exercise (both for day 1 and day 2), while decreasing in participants with ME/CFS for the same period (apart from data for day 2 women). The observed decrease is even more pronounced in male participants.

None of the changes between female participants and controls for those 11 metabolites were significantly different between D1PRE and D2PRE (*q* > 0.75), meaning that their levels were back to baseline after 24 hours of recovery. They were also not statistically different between D2PRE and D2POST (*q* > 0.75), meaning that the initial differences caused by exercise between controls and patients at day 1 were not repeated on day 2. Out of these same 11 metabolites, only pyruvate (*q* = 0.01), lactate (*q* = 0.01), and hypoxanthine (*q* = 0.13) were significantly different when considering D1PRE and D2POST, reflecting changes happening after 2 maximal CPETs over 24 hours. Except for hypoxanthine and the dipeptide isoleucyl-glycine, 9 out of the 11 metabolites were significantly different when considering the 24-hour recovery period (D1POST to D2PRE).

When sorting each column in Supplemental Table 6 and counting the metabolites with statistically different profiles between controls and patients (*q* < 0.15), D1PRE to D2PRE adds up to 15 metabolites, D1PRE to D2POST to 12 metabolites, D1POST to D2PRE to 61 metabolites, D1POST to D2POST to 14 metabolites, and D2PRE to D2POST to 15 metabolites. None of these metabolites were significantly different for all comparisons. Eight of them were significant for several comparisons (*q* < 0.15), except for D1PRE to D1POST. Those metabolites included 3 unknowns, 2 “amino acids” (proline and threonine), 2 “lipids” (adipoylcarnitine [C6-DC] and 2R,3R-dihydroxybutyrate), as well as 1 “xenobiotic (4-vinylphenol sulfate).

In the category with the most metabolite behavior differences (D1POST to D2PRE), 14 out of 61 (23%) were unknowns (including 15245, Figure 11A; as well as the energy metabolite alpha-ketoglutarate, Figure 11B), 20 of the 47 identified metabolites were “amino acids” (43%), 15 were “lipids” (32%), 4 were “carbohydrates” (9%), 5 were “xenobiotics” (11%), and 1 was “cofactor and vitamins”: pantothenate (vitamin B_5_).

The 2-fold enrichment of the “amino acid” superpathway metabolites (23% of known metabolites are amino acids) could indicate a differential rebalancing of amino acids during the 24-hour recovery period in participants with ME/CFS compared with controls, especially from the “leucine, isoleucine and valine metabolism” as well as the “arginine and proline metabolism” pathway. Half of those amino acids were significant solely for the D1POST to D2PRE period, and an additional 5 had only 1 other significant time point, D1PRE to D1POST.

Concerning the lipids, 8 out of the 15 (53%) contain carnitine, all with *q* < 0.04 and chains shorter than 12 carbons. Although other short- and medium-chain carnitines also had nonsignificant differences between controls and patients, none of the long-chained carnitines were significant at D1POST to D2PRE. Carnitine lipids represented 6% of our data set and 20% of the lipids but 17% of the significant metabolites at D1POST to D2PRE and 53% of the lipids.

### Many of the most altered metabolites contain carnitine.

One of the modules of MetaboAnalyst supports varying time-series approach, including multivariate analysis, such as the Multivariate Empirical Bayes Analysis of Variance (MEBA). Supplemental Table 7 is available for further exploration of the complex output beyond what is discussed below. It includes each individual cohort (total, women, and men) for controls and patients, as well as the controls/patients combined comparison, for a total of 9 columns and over 10,000 Hotelling *T*^2^ tests.

**Figure 12:**
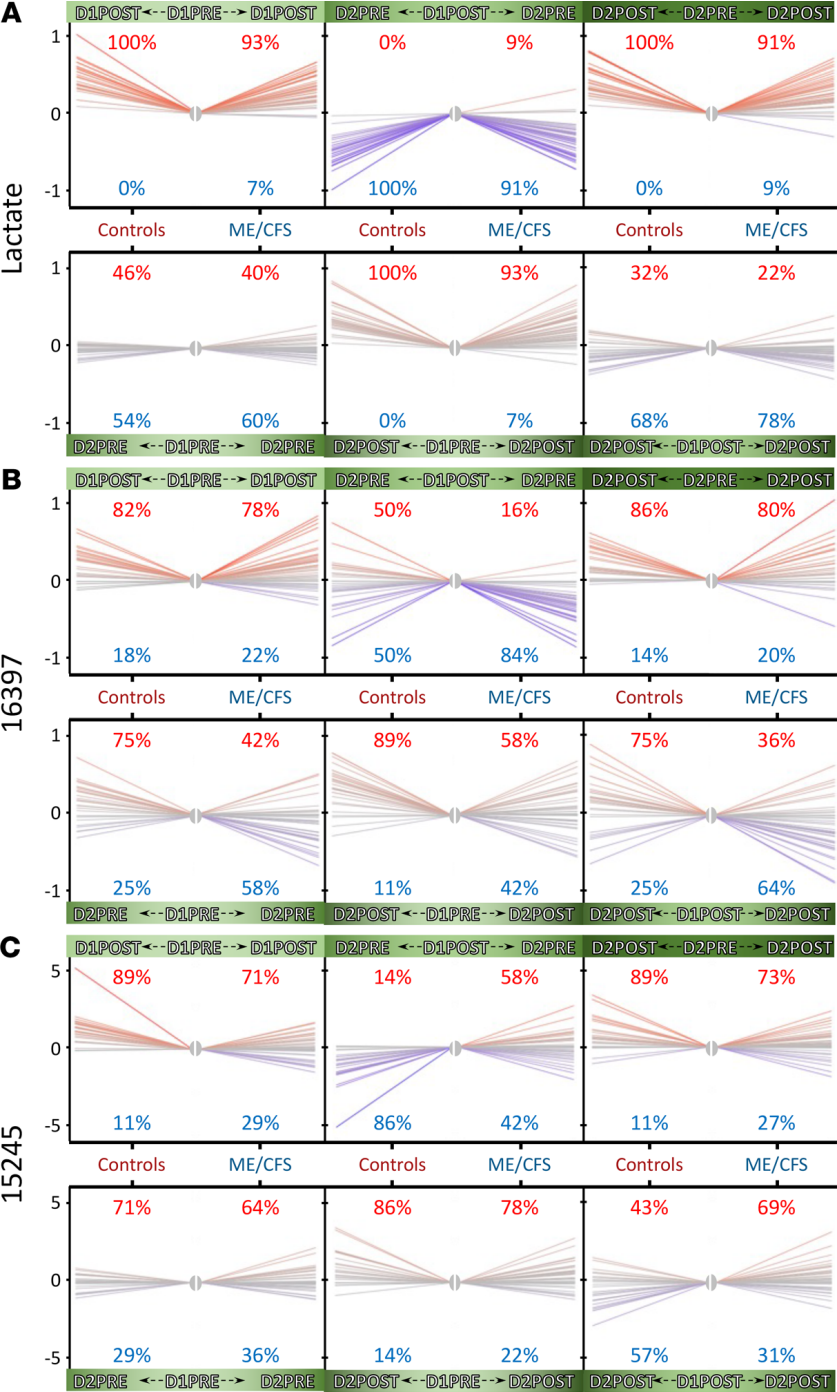
Spaghetti plots showing the amplitude of the variations for selected metabolites between all 4 time points for controls versus patients. (**A**) Lactate. (**B**) 16397. (**C**) 15245.

The highest Hotelling *T*^2^ value was 419 for lactate within the female control cohort, which means that this metabolite incurred the most changes of all 1157 metabolites during this experimental protocol. The corresponding Hotelling *T*^2^ value for female participants with ME/CFS was 150, ranking fifth after malate (*T*^2^ = 293), 3-methyl-2-oxobutyrate (*T*^2^ = 258), 4-methyl-2-oxopentanoate (*T*^2^ = 244), 3-methyl-2-oxovalerate (*T*^2^ = 209), and pantothenate/vitamin B_5_ (*T*^2^ = 163). Those 5 metabolites also ranked high in the control cohort Hotelling *T*^2^ scores. Figure 12A displays the plasma lactate changes for each female participant throughout the 4 time points. We noticed similar patterns between controls and participants with ME/CFS where lactate increased during both maximal CPETs (D1PRE to D1POST panel and D2PRE to D2POST panel). During the 24-hour recovery period (D1POST to D2PRE panel), lactate amounts decreased. In the D1PRE to D2PRE panel, we observed a consistency of lactate amount in the blood between the baseline value and after 24 hours of recovery. Comparatively, the D1POST to D2POST panel shows slopes that tend to be more negative, reflecting a lesser increase of plasma lactate during the second maximal CPET compared with the first one. Finally, the D1PRE to D2POST panel reflects the state of the participants immediately after the complete course of this study compared with their original baseline, with a similar pattern as the D1PRE to D1POST and D2PRE to D2POST panels for lactate.

When sorting the female control/patient Hotelling *T*^2^ scores by descending values, the highest value, of 43, was for a compound of unknown identity, 16397. The fluctuations for this metabolite were different than the ones described for lactate (Figure 12B). Moreover, there were clear differences between controls and participants with ME/CFS. For instance, during the 24-hour recovery period (D1POST to D2PRE panel), the amounts of plasma metabolite 16397 went down in 84% of the patients, while 50% behaved similarly in controls. The 3 bottom panels also have distinct percentages between controls and patients, especially when comparing baseline and 24-hour recovery (D1PRE to D2PRE panel) as well as the D1POST and D2POST panel, with inverted percentage patterns.

Another compound of unknown identity, 15245 (also mentioned in Figure 11A), had an elevated Hotelling *T*^2^ score of 39. This metabolite had a reasonably similar pattern between controls and patients for most panels (Figure 12C), apart from the 24-hour recovery period (D1POST to D2PRE panel), where the amount of plasma 15245 decreased in controls but increased in more than half (58%) the patients. This pattern was amplified when examining the D1POST to D2POST panel, where plasma 15245 increased in 69% of the patients.

Although each metabolite had a distinctive pattern, by examining the female control/patient Hotelling *T*^2^ scores higher than an arbitrary value of 15, we determined that 16 out of 65 metabolites (25%) were unknown. Of the remaining 49, 13 out of 20 lipids were carnitine-containing compounds (65%); 6 out of 14 amino acids were part of “leucine, isoleucine and valine metabolism” (43%); 3 were “carbohydrates,” namely pyruvate, 1,5-AG, as well as lactate; and 1, alpha-ketoglutarate, was part of the “energy” superpathway.

A similar implementation with the male control/patient Hotelling *T*^2^ scores that were higher than 15 produced 19 metabolites, with 2 of unknown identity (10%); 1 carnitine-containing lipid out of 9 lipids (10%); and gluconate as a metabolite part of the “energy” superpathway (Supplemental Table 7).

## Discussion

Historically, ME/CFS blood metabolomics studies have been observations of the baseline status of the participants, and our study design also allows such analysis (D1PRE data). Two recent reviews (23, 24) organized the ME/CFS metabolomics literature to assess consistency between groups and measurement methods used. When the reviews focused on reproducibility, the pathways that were most consistent between studies included amino acid metabolism and urea cycle, glycolysis, lipid metabolism, and redox-related pathways. It is important to reaffirm that disparities in the methods used affected the number of metabolites assessed with a strong impact on pathway output estimates. Notably, earlier studies were limited to just over 20 metabolites, whereas contemporary ones range from 144 (12) to 832 (11) or 880 (20), and even 933 identified metabolites for this latest data set (1157 when including the unknowns).

Most of the metabolites significantly different between participants with ME/CFS and controls in the female cohort had lower means in patients, with only 1 higher at *q* < 0.05 (indoleacetoylcarnitine) out of the 7 (Figure 1 and Supplemental Table 4). At both *q* < 0.15 and *P* < 0.05, only 25% had higher means (Supplemental Table 4). Nevertheless, out of the 1157 plasma metabolites assessed, we did not identify any discriminatory metabolite or group of metabolites that could serve as a biomarker on its own. However, the identification of 1,5-AG as one of the few highly significantly different features of our data set and its patented use to monitor short-term glycemic control directs attention to energy metabolism as do the pathway analysis results (Figure 6). Unlike in our first study where glucose appeared to be reduced in patients (10), glucose here was slightly elevated in our patient cohorts, consistent with our second study (11).

Fluctuations between studies and between subjects within a study are often argued as resulting from ME/CFS symptom variations, from outside factors, or as the proof for a need to seek and establish subgroups (17, 20). Certainly it is possible that subgroups exist, but describing them after inspection of the data can lead to invalid conclusions because of HARKing (25). Large-scale cohorts combined with large-scale untargeted metabolomics are needed, permitting the dissociation of individual metabolic environments (depicted in Figure 2) from the disease metabotype.

### Sex differences in metabolomes of participants with ME/CFS.

Subgroups that can easily be hypothesized before viewing any sort of molecular data are women versus men. ME/CFS is a female-dominant disease; an estimated 25% to 35% of the patients affected are men (1). Due to the difficulty in obtaining sufficient numbers of men in small-scale studies, including some of our prior ones (9–11), exclusively female cohorts are sometimes selected (13–15). Other studies, which have approximately 20% men and small size cohorts (50 patients or less), acknowledge the lack of power to test sex disparities (12, 17, 19). In 2016, a cohort evenly split between sexes, gathered by Naviaux et al. (18), revealed that the metabolic features of women and men have some commonalities as well as many differences in the pathways ME/CFS affects. Using another evenly split cohort, Nkiliza et al. (26) observed sex-specific effects on lipid metabolites. In a cohort of 200 participants with 20% men (16), trends were distinctive between sexes but fell short in statistical significance for the male component. A recent study using a plasma Metabolon data set of 880 serum metabolites from a cohort of 83 participants with ME/CFS and 20% men did not separate women from men. Instead, a correlation analysis separated patients into metabotypes unrelated to sex (20).

Because female participants compose 71% of our cohort, most of the significant results depicted in the figures focus on female patterns. Nevertheless, we strived to exploit the data derived from the limited number of male participants by drawing comparisons in most analyses, while acknowledging the reduced size of the male cohort. Figures 3, 4, 5, and 11; Supplemental Figure 6; as well as various other nonillustrated results lay out converging evidence of sex-specific metabolic patterns.

A unique feature of our ME/CFS study is the exercise component that includes a majority-female patient cohort. Blood metabolomics studies of exercised patients have been carried out since the 1970s (27–30), but these studies were male dominant, especially when athletes were recruited. An elegant multiomic study of response to exercise of healthy individuals, where 58% of the 36 participants are men, was compiled without sex specificity (8).

### Unidentified metabolites may play a critical role in ME/CFS.

One aspect of this Metabolon data set is the compilation of metabolites yet to be identified. We requested this extra information to weigh the importance of unknowns in our quest to elucidate the mechanisms of ME/CFS, which might serve to motivate future studies. Our data demonstrate an enrichment of unknown metabolites for higher statistical thresholds (25% to 43% depending on the time point, while composing 19% of the original data set). The prevalence of such a category certainly has far-reaching consequences in our ability to decode ME/CFS and its complex features. At the time of writing, we know that 07765 (Figure 4) is possibly a sulfated phenol, 23680 (Figure 4) is possibly a fatty acid carnitine, 18921 (Figure 4) appears to be acetylated, and 15245 (Figures 11 and 12) is possibly an acetylated organic acid. Although such limitations are part of the field of metabolomics, this information is helpful, as it brings up the percentage of carnitine compounds that are significantly different at *q* < 0.05 for D1PRE to 29% (2 out of 7).

### Several pathways previously noted to be altered in ME/CFS also appear in our study.

Fatty acid metabolites have previously been noted to be at different levels in ME/CFS and control plasma (9, 10). Carnitine is an ammonium compound essential for fatty acid oxidation, and both “oxidation of branched-chain fatty acids” and “carnitine synthesis” were affected in ME/CFS patient pathway analysis (Figure 7). The oxidation of branched-chain fatty acids is necessary to produce an even-numbered carbon chain that can then enter fatty acid oxidation and produce acetyl-CoA, a source of energy in the heart and brain. “Phytanic acid peroxisomal oxidation” is another important component of fatty acid intake.

The Contrepois et al. study of healthy individuals (8) assessed metabolites from the TCA cycle and found them increased at both 15 and 30 minutes after exercise. The enhanced increase in ME/CFS cases (Figure 7) most likely reflects some compensation for a lack of alternative energy sources. A shift in amino acid metabolism is also observed in Figure 7, especially for alanine, aspartate and glutamate, arginine, and proline metabolism. The levels of most of those amino acids were found to be reduced after exercise in healthy participants (8). Armstrong et al. (13) previously reported altered amino acid and nitrogen metabolism in ME/CFS. Another category of pathways in our study is related to nitrogen metabolism, including “urea cycle” and “ammonia recycling,” in which glutamate and glutamine also play an important role according to SMPDB curation.

Many of the highly significant pathways in Figure 8 are related to energy metabolism, including amino acid metabolism, sugars, “citric acid cycle,” “pyruvate metabolism,” and “glycolysis.” Such pathways are familiar from the metabolomics ME/CFS literature (23, 24), but their evolution through the recovery period is probably novel. The differences of recovery of these key energy pathways illustrate the effect of ME/CFS on those pathways. Among altered pathways is one labeled “Warburg effect,” which refers to the mode of energy production of cancer cells, where glucose is preferentially used to produce lactate (aerobic glycolysis). We cannot know from our data whether alteration in this pathway might be due to differential levels of hypoxia.

Glutamate metabolism is central to many of the pathways disturbed in ME/CFS. Out of all the significantly different pathways in the ME/CFS panel output of Figure 7, “glutamate metabolism” caught our attention, due to its wide-ranging roles in numerous metabolic pathways, including many among the highlighted pathways of Figure 7. Apart from being an amino acid precursor to many nucleic acids and proteins, glutamate also plays a role in the central nervous system as an excitatory neurotransmitter; it affects memory and learning through the modulation of neuronal plasticity. According to the curated SMPDB description, disorders resulting from a dysfunctional glutamate metabolism affect ammonia levels (hyperammonemia), brain activity (γ-hydoxybutyric aciduria), red blood cells (hemolytic anemia), and amino acid metabolism (5-oxoprolinuria).

The SMPDB description of several of the other pathways in Figure 7 involves glutamate, including “butyrate metabolism,” in which molecules are produced that are precursors for glutamate synthesis. We previously reported lower levels of butyrate-producing bacteria in the gut microbiome of individuals with ME/CFS (31, 32). Glutamate is an intermediate precursor in “arginine and proline metabolism.” In “citric acid cycle,” alpha-ketoglutarate can serve as a precursor to glutamate. Glutamate is produced during “lysine degradation.” “Glucose-alanine cycle” involves muscle protein degradation and glutamate as an intermediate transported to the liver, where it is funneled into gluconeogenesis for glucose to be transported back to muscles for ATP production. “Alanine metabolism” is involved in pyruvate conversion. Glutamate can also be catabolized into ammonium in the mitochondria and then enters the “urea cycle.”

We note that neither glutamate nor glutamine itself was statistically different between controls and participants with ME/CFS at D1PRE (*P* = 0.4 and *P* = 0.9 respectively) and throughout the time points (Supplemental Table 4). Lack of differential abundances of 1 or 2 components of a pathway does not mean that the pathway itself is not altered between 2 groups of participants. Instead, glutamate is a common denominator of the pathways significantly affected by exertion in participants with ME/CFS. Glutamate variations have been associated with ME/CFS before but not always with reproducibility. Indeed, while glutamate was slightly decreased in our previous female cohort (9), it was actually slightly increased in the plasma of both women and men in this data set. Armstrong et al. reported significant reduction in glutamate amounts (14), specifically in women and not in men according to Fluge et al. (16). On the other hand, Naviaux et al. noted normal levels of glutamate in both their female and male cohorts (18).

The literature on glutamate-related metabolism is extensive and addresses all aspects of glutamate’s numerous roles in the homeostasis of the body.

Although all the changes we have observed exist in the plasma, blood is a system directly in contact with all other systems of the body. Dysfunctions in glutamate metabolism affect inflammation in the brain (33–35), many neurological diseases including epilepsy and Alzheimer’s (36–38), the microbiome (39, 40), the immune system (41), and redox status (42), to mention a few examples. However, glutamate/glutamine was not found to be altered when its level was assessed by neuroimaging of participants with ME/CFS and controls (43). This finding is consistent with the lack of altered levels in plasma, though it cannot be assumed that plasma levels of a metabolite reflect its abundance in particular tissues and organs.

### The recovery period of participants with ME/CFS is highly disrupted compared with healthy controls.

Few studies investigate metabolomics evolution during the recovery period after a brief but intense exercise, and the ones that do focus on male athletes, where they find metabolite shifts return to baseline levels after 24 hours (44).

The major trend displayed in Figure 8 and Figure 10, A and E, shows that over the 24-hour recovery period, an increasing number of significant metabolic disturbances accumulated between participants with ME/CFS and controls. This trend is also noticeable when comparing the panels in Supplemental Figure 2 as well as between Figure 9 and Supplemental Figures 3–5, where most of the original clusters shown in blue remain constant throughout the 4 figures, while the ones that become significant are depicted mainly as red or purple, reflecting a shift from hypometabolism of significant metabolites toward hypermetabolism for newly significantly different metabolites. An association between PEM and hypermetabolism has already been inferred in ME/CFS in an uncontrolled PEM induction setting (19).

Our longitudinal study design has allowed us to identify a number of pathways that diverge between healthy individuals and those with ME/CFS 24 hours after an exercise challenge, at which time patients typically experience PEM. Inability to recover properly after exertion is one of the most disabling symptoms of ME/CFS. Our study provides insight into the metabolic changes that are inimical to proper response to physical effort.

## Methods

### Cohort selection.

A total of 105 participants, subdivided between 60 participants with ME/CFS (45 women plus 15 men) who fulfilled the Canadian consensus criteria for ME/CFS diagnosis (45) and 45 controls (30 women plus 15 men), underwent 2 successive CPETs at 3 sites. Participants were identified as ME/CFS cases or healthy sedentary controls by medical doctors and tested at Ithaca College, Weill Cornell Medicine, and Workwell Foundation.

### Exercise protocol and blood collection.

Samples were collected before and after 2 maximal-effort CPETs on a stationary cycle that were separated by a 24-hour recovery period. The protocol was a 15-Watt ramp increment every 1 minute until the participant could no longer maintain a cadence of at least 50 rpm. The duration of cycling to maximal effort varied by participant from 6 to 14 minutes. Patients and corresponding healthy but sedentary participants were exposed to the same exercise protocol to allow statistical comparison. Four longitudinal blood samples were drawn from a vein in the antecubital fossa into EDTA tubes and conveyed to processing laboratories the same day. Plasma was isolated following centrifugation of blood at 500*g* for 5 minutes at room temperature. Plasma samples were immediately stored at –80°C.

### Metabolomics data set.

Plasma samples thawed once for aliquoting were shipped on dry ice to Metabolon’s facility in North Carolina (https://www.metabolon.com), where the Precision Metabolomics LC-MS global metabolomics platform was carried out to provide a spreadsheet data output of relative concentrations. An initial step undertaken was to populate the missing values by finding the minimum value for each metabolite and imputing that value to any missing value within that metabolite. The exception was for drug-related and tobacco-related metabolites, for which a 0 was used for imputation, as the missing values were likely due to true absence rather than instrument detection limits.

When the tools described in the next section required all 4 time points, some participants had to be excluded because some blood draws were not possible, especially after exercise. For those analyses, the female cohort consists of 28 controls and 45 participants with ME/CFS while the male cohort consists of 14 controls and 10 participants with ME/CFS.

### Tools used for data organization.

Participants provided demographic data and completed the Bell scale and SF-36 questionnaires, which were organized using REDCap (https://www.project-redcap.org).

### Statistics.

R scripts were used to perform Wilcoxon’s rank-sum tests, to perform the t-SNE analysis, and to draw box plots and spaghetti plots. The original Venn diagram analyses were done using http://bioinformatics.psb.ugent.be/webtools/Venn The MetaboAnalyst 5.0 modules used throughout this manuscript were carried out with the online tool at https://www.metaboanalyst.ca (46). MetaboAnalyst is an open-source suite of analytic tools provided by the Xia lab since 2009 (47), with an increasing number of analytic modules with each new version (48–50). Each module relies on established models developed by varying groups and mathematical units. For instance, the MEBA we applied uses the Hotelling *T*^2^ test, where the null hypothesis represents consistency in time and/or between cohorts, based on the work by Tai et al. (51). The higher the ranking score, the higher the differences between temporal points or cohorts. The pathway analysis integrates pathway enrichment analysis and pathway topology analysis. The ChemRICH analyses were performed online at http://chemrich.fiehnlab.ucdavis.edu by providing the required input files (52). ChemRICH is described by its founders as “a statistical enrichment approach based on chemical similarity” rather than traditional pathway analysis approaches, which rely on previous knowledge of the involvement of a metabolite in a given pathway. Other simple calculations were done in Microsoft Excel. Supplemental Figure 1 was drawn using JMP Pro 16.0.

When thousands of hypothesis tests, in this case Wilcoxon’s rank-sum tests, are conducted at the same time, the probability for metabolites to be wrongfully called significantly different at *P* < 0.05, also known as false positives, increases proportionally to the number of tests conducted. Mathematically, this statistical error requires correction by controlling for the FDR. We used the Benjamini-Hochberg procedure to calculate *q* values for each series of tests performed. On the other hand, the relevance of arbitrary cutoffs has been a subject of debate when interpreting biological data sets (53). Overcorrection will inevitably result in false negatives and hinder the biological interpretation of the significantly different features. We designed our presentation of the data around the pursuit of transparency and display 3 cutoffs from the most conservative (*q* < 0.05), to a slightly less conservative one (*q* < 0.15), and finally with no correction for multiple testing (*P* < 0.05). This allows for flexibility in our data interpretation and trend discovery as well as leniency for our size-limited male cohort.

### Study approval.

Protocols regarding human participants were approved by the Institutional Review Boards of Weill Cornell Medical College (Protocol 1708018518) and Ithaca College (IRB 1017-12Dx2). Written informed consent was received prior to participation.

## Author contributions

MRH, A Grimson, and BAK designed the study. GEM, SML, and JKC identified appropriate participants. GEM monitored participant safety. BAK and JS performed CPETs. SML, JKC, BAK, JS, CJF, XM, and DCS supervised collection and/or use of plasma samples and clinical data. A Germain, LG, and MRH interpreted metabolomics data. A Germain and MRH wrote the manuscript. A Germain, MRH, and A Grimson edited the manuscript. All authors read and approved the manuscript.

## Supplementary Material

Supplemental data

Supplemental data set 1

Supplemental table 4

Supplemental table 5

Supplemental table 6

Supplemental table 7

## Version 1 03/31/2022

In-Press Preview

## Version 2 05/09/2022

Electronic publication
