# Supplementary material for: Plasma metabolomics reveals disrupted response and recovery following maximal exercise in myalgic encephalomyelitis/chronic fatigue syndrome
Source: JCI Insight. 2022 May 9;7(9):e157621. doi: 10.1172/jci.insight.157621 (PMC9090259; doi:10.1172/jci.insight.157621)
Supplement: Supplemental data [file jciinsight-7-157621-s148.pdf]

Supplementary Material

Germain et al. 2022

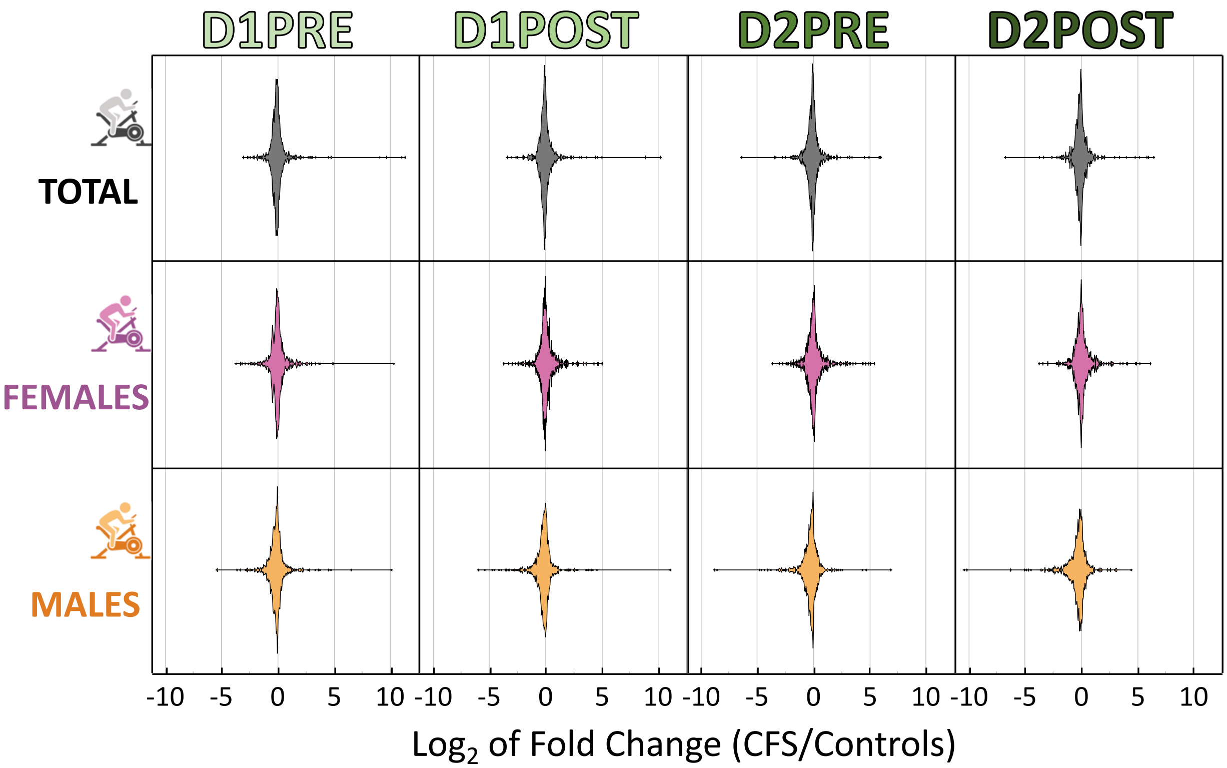

Supplementary Figure 1: Violin plots of the $\log_2$FC distribution for each timepoint and each cohort.

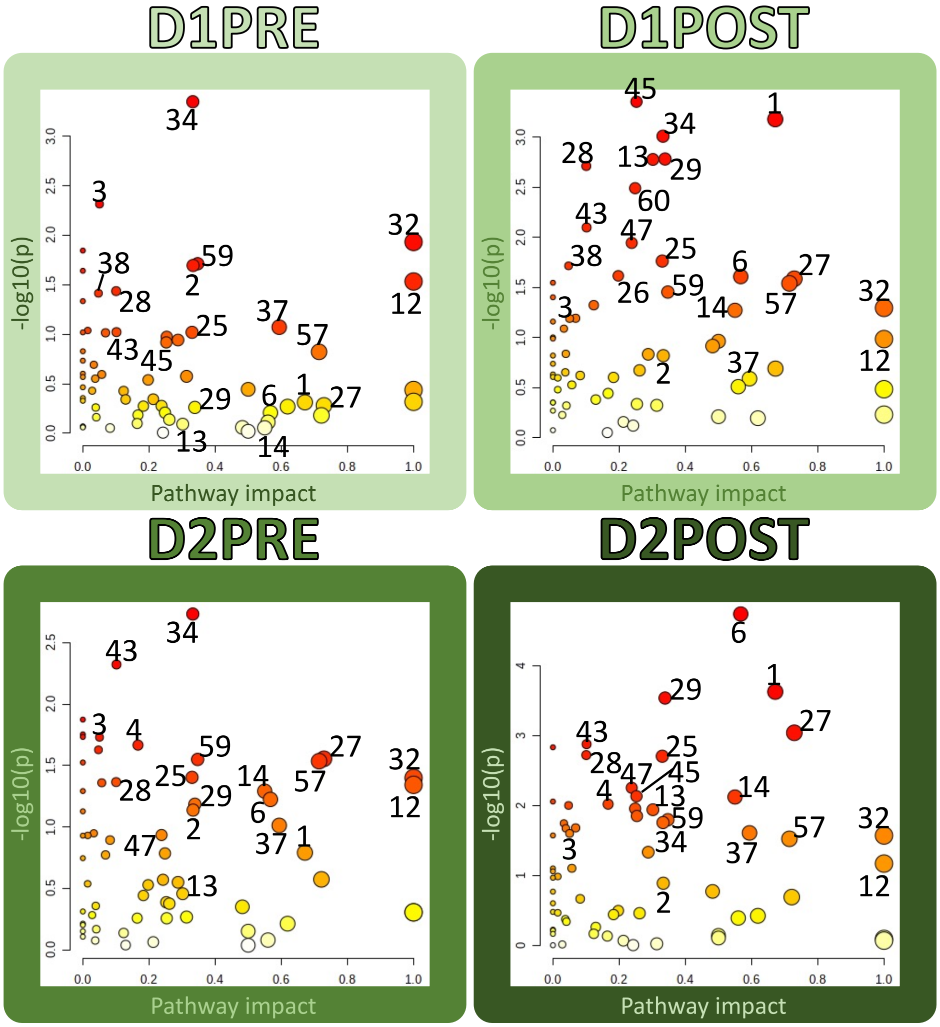

Supplementary Figure 2: Pathway analysis results from MetaboAnalyst at each timepoint for the female cohort. The pathway impact on the y-axis from 0 (low impact) to 1 (strong impact), represents the values from the pathway topology analysis. Each circle denotes a pathway, and the fill-in color represents the significance of disturbances in that pathway from white (low significance) to red (higher significance). The pathway numbers are as follows: #1 “Alanine, aspartate and glutamate metabolism”; #2 “alpha-Linolenic acid metabolism”; #3 “Amino sugar and nucleotide sugar metabolism”; #4 “Aminoacyl-tRNA metabolism”; #6 “Arginine and proline metabolism”; #12 “Caffeine metabolism”; #13 “Citrate cycle (TCA cycle)”; #14 “Cysteine and methionine metabolism”; #25 “Glycerolipid metabolism”; #26 Glycerophospholipid metabolism”; #27 “Glycine, serine and threonine metabolism”; #28 Glycolysis/Gluconeogenesis”; #29 “Glyoxylate and dicarboxylate metabolism”; #32 “Linoleic acid metabolism”; #34 “Nicotinate and nicotinamide metabolism”; #37 “Pentose and glucuronate interconversions”; #38 “Pentose phosphate pathway”; #43 “Primary bile acid biosynthesis”; #45 “Purine metabolism”; #47 “Pyruvate metabolism”; #57 “Taurine and hypotaurine metabolism”; #59 “Tryptophan metabolism”; #60 “Tyrosine metabolism”.

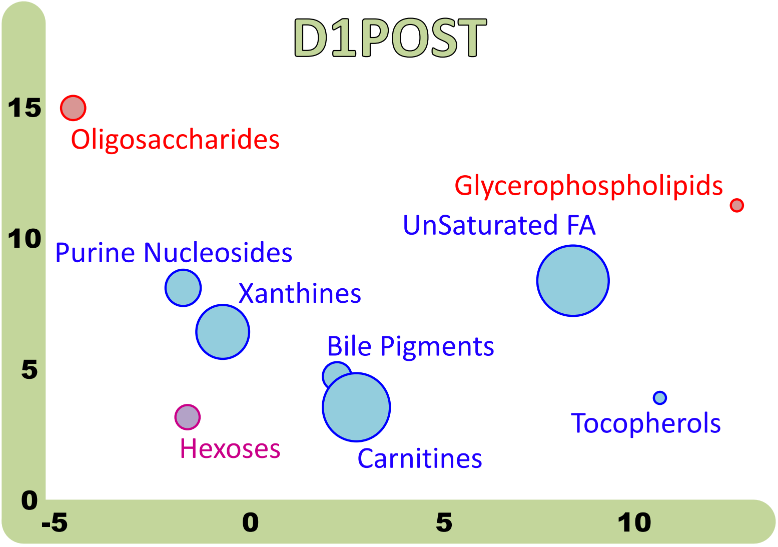

Supplementary Figure 3: ChemRICH output for D1POST for the female cohort. Only clusters enriched at $p < 0.05$ are shown. The x-axis is the cluster order on the similarity tree. The y-axis is the -log(p-value), with the most significantly altered clusters at the top. The color scale represents the portion of metabolites with a ratio of patients/controls either decreased (in blue), increased (in red) or an equal number of metabolites both increased and decreased (in purple).

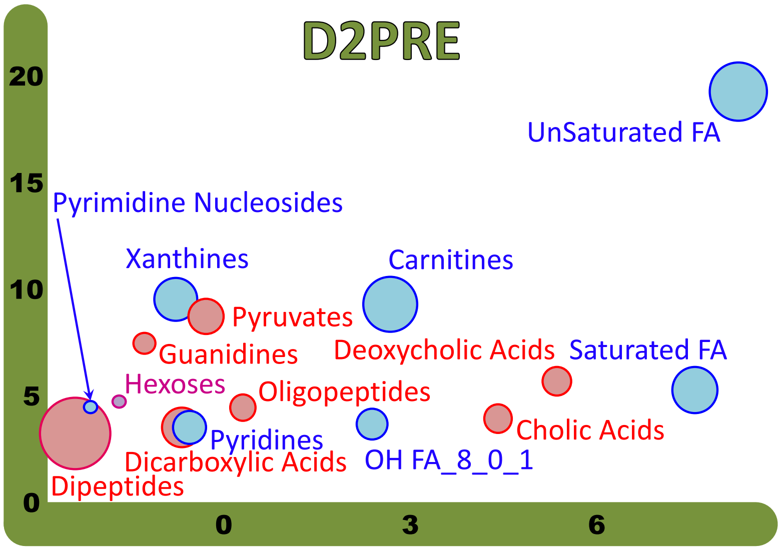

Supplementary Figure 4: ChemRICH output for D2PRE for the female cohort. Only clusters enriched at $p < 0.05$ are shown. The x-axis is the cluster order on the similarity tree. The y-axis is the -log(p-value), with the most significantly altered clusters at the top. The color scale represents the portion of metabolites with a ratio of patients/controls either decreased (in blue), increased (in red) or an equal number of metabolites both increased and decreased (in purple).

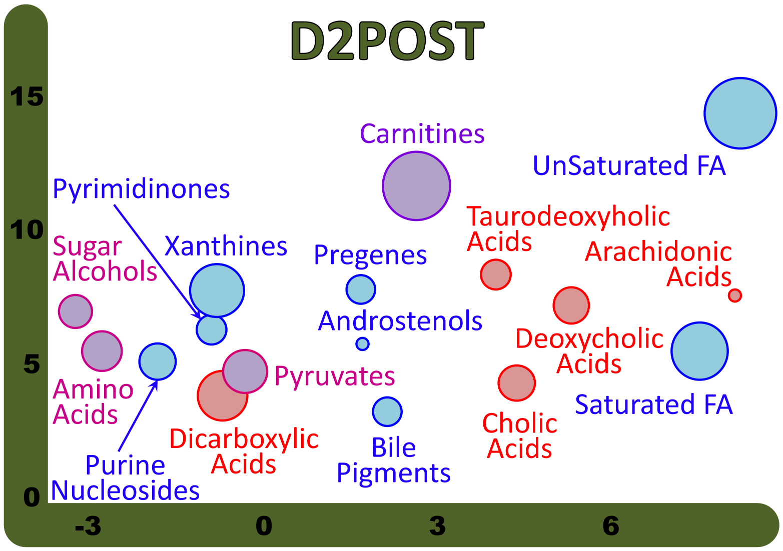

Supplementary Figure 5: ChemRICH output for D2POST for the female cohort. Only clusters enriched at $p < 0.05$ are shown. The x-axis is the cluster order on the similarity tree. The y-axis is the -log(*p*-value), with the most significantly altered clusters at the top. The color scale represents the portion of metabolites with a ratio of patients/controls either decreased (in blue), increased (in red) or an equal number of metabolites both increased and decreased (in purple).

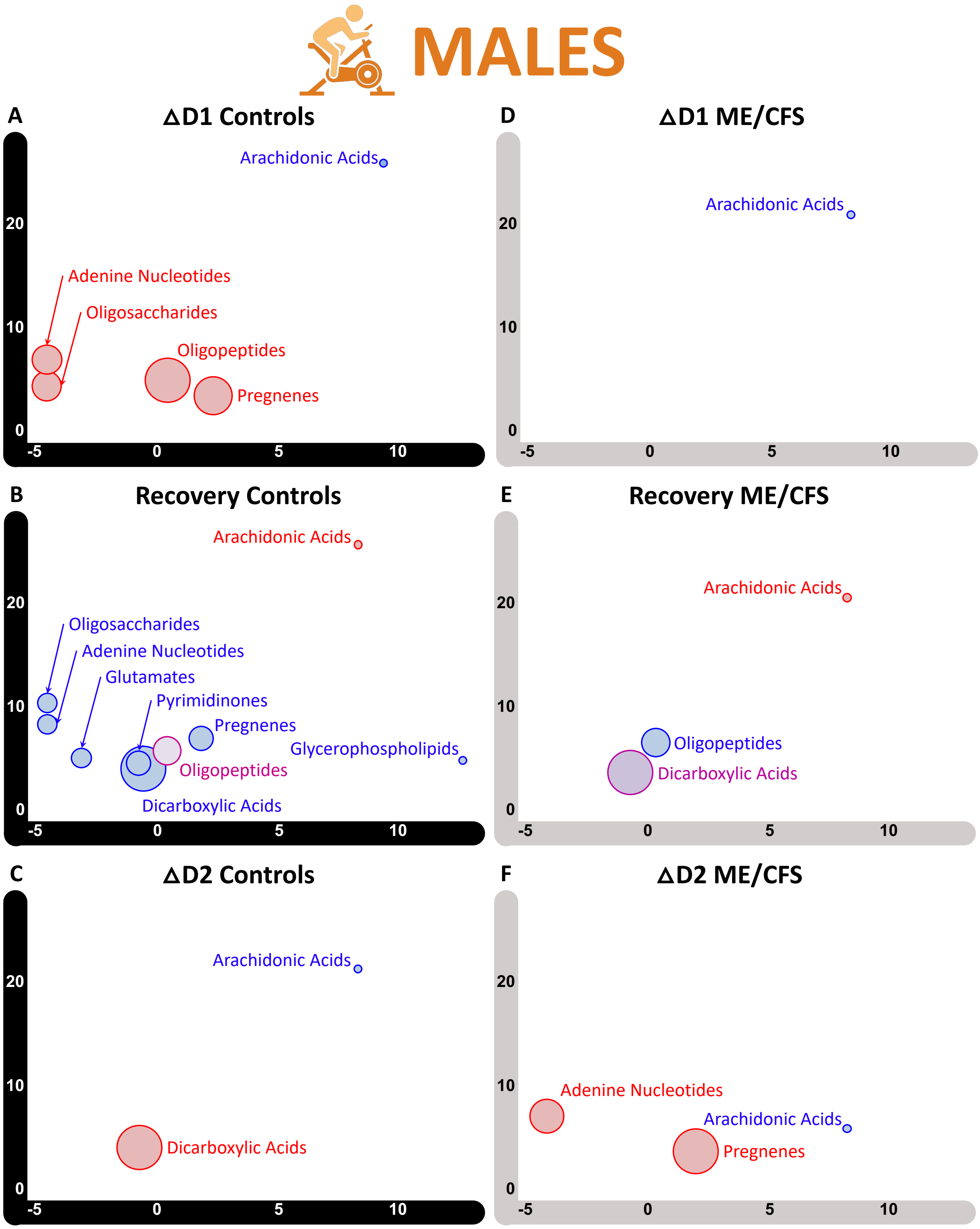

Supplementary Figure 6: ChemRICH output of ΔD1, the 24-hour recovery period, and ΔD2 for male controls and ME/CFS patients. Only clusters enriched at $p < 0.05$ are shown. The x-axis is the cluster order on the similarity tree. The y-axis is the -log(p-value), with the most significantly altered clusters at the top. The color scale represents the portion of metabolites with a ratio of patients/controls either decreased (in blue) increased (in red) or an equal number of metabolites both increased and decreased (in purple).

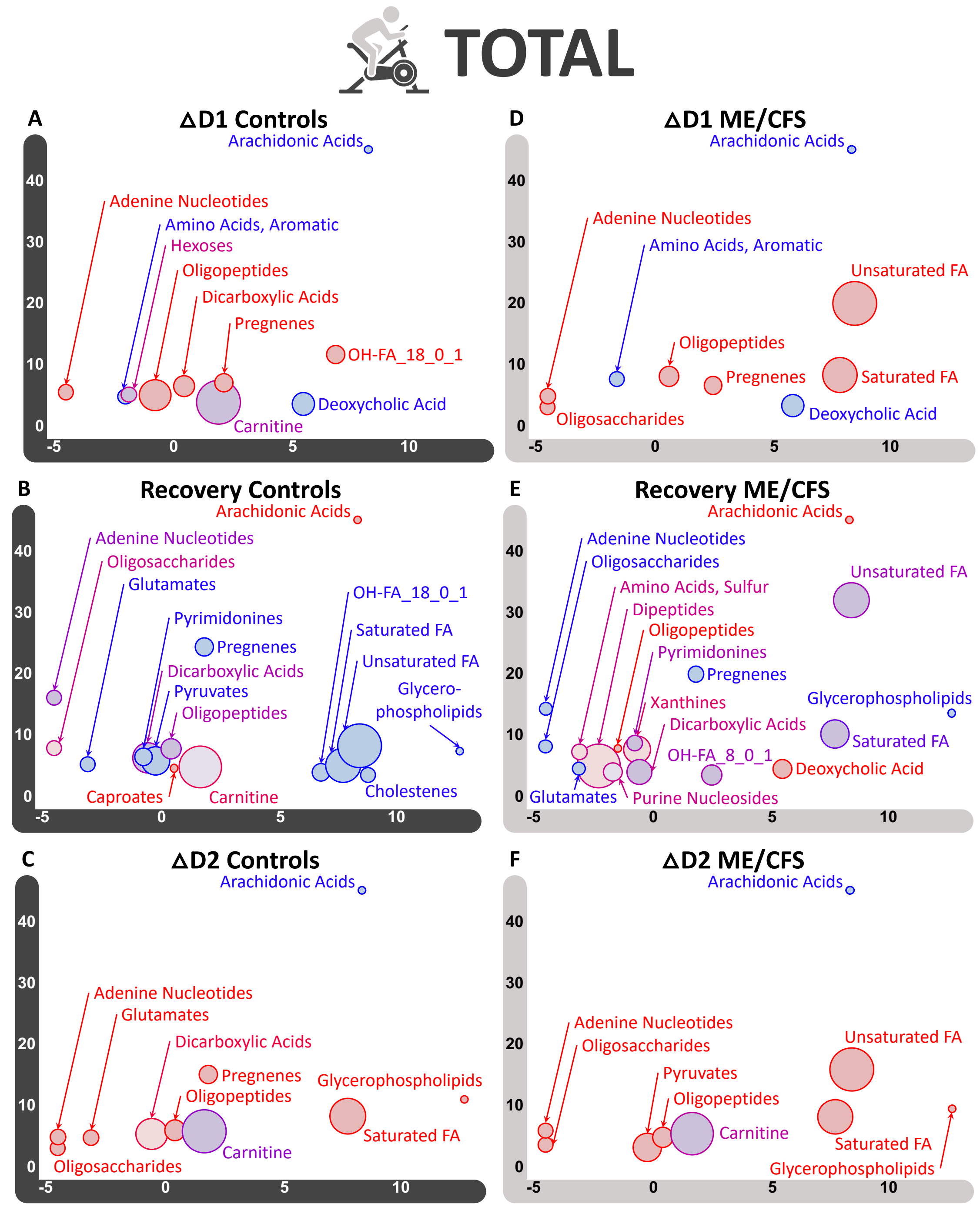

Supplementary Figure 7: ChemRICH output of ΔD1, the 24-hour recovery period, and ΔD2 for all controls and ME/CFS patients. Only clusters enriched at $p < 0.05$ are shown. The x-axis is the cluster order on the similarity tree. The y-axis is the -log(p-value), with the most significantly altered clusters at the top. The color scale represents the portion of metabolites with a ratio of patients/controls either decreased (in blue) increased (in red) or an equal number of metabolites both increased and decreased (in purple).

Supplementary Table 1. Details of the population statistics without sex segregation.

		Controls n = 45	ME/CFS n = 60
**Sex (n)**	Female	30	45
	Male	15	15
**Ethnicity**	Hispanic or Latino	13%	17%
	Not Hispanic or Latino	82%	78%
	ND	4%	5%
**Race**	American Indian or Alaska native	2%	0
	Asian	11%	5%
	Black or African American	4%	0
	Native Hawaiian or other Pacific Islander	0	2%
	White	76%	88%
	ND	7%	5%
**Age**	Mean +/- SD	42.6 +/- 13.5	46.7 +/- 11.5
	Median (min-max)	44 (18-66)	48.5 (21-69)
**BMI**	Mean +/- SD	28.2 +/- 5.4	26 +/- 5.3
	Median (min-max)	26.8 (20.6-41.8)	25.9 (18.5-39.1)
**Illness duration (years)**	Mean +/- SD	NA	11.7 +/- 9.8
	Median (min-max)		8 (1-38)
**Type of onset**	Gradual	NA	43%
	Sudden		55%
	ND		2%
**Bell's disability scale***	10-20	0	15
	30-40	0	36
	50-60	1	8
	70-80	2	1
	90-100	42	0
**SF-36***	Physical Component Summary **(PCS)**	55.9 +/- 4.6	28.2 +/- 7.2
	Mental Component Summary **(MCS)**	55.1 +/- 5.1	44.2 +/- 10.2

* Higher scores represent better health.
NA: Not applicable.
ND: Not determined.

Supplementary Table 2. Details of the population statistics with sex segregation.

		Controls (n = 45)		ME/CFS (n = 60)	
		Female	Male	Female	Male
**Sex (n)**		30	15	45	15
**Ethnicity**	Hispanic or Latino	17%	7%	13%	27%
	Not Hispanic or Latino	80%	87%	80%	73%
	ND	3%	7%	7%	0
**Race**	American Indian or Alaska native	3%	0	0	0
	Asian	10%	13%	7%	0
	Black or African American	3%	7%	0	0
	Native Hawaiian or other Pacific Islander	0	0	2%	0
	White	73%	80%	89%	87%
	ND	10%	0	2%	13%
**Age**	Mean +/- SD	42.1 +/- 14.5	43.5 +/- 11.8	47.4 +/- 11.8	44.7 +/- 10.8
	Median (min-max)	43 (18-66)	46 (24-64)	50 (21-69)	47 (23-58)
**BMI**	Mean +/- SD	28.8 +/- 5.8	26.9 +/- 4.6	25.2 +/- 5.4	28.2 +/- 4.6
	Median (min-max)	27.4 (20.6-41.8)	25.8 (21.4-37.5)	24.4 (18.5-39.1)	26.8 (19.8-38.4)
**Illness duration (years)**	Mean +/- SD	NA	NA	11.2 +/- 9.6	13.3 +/- 10.6
	Median (min-max)			7 (1-38)	10 (1-36)
**Type of onset**	Gradual	NA	NA	42%	
	Sudden			56%	
	ND			2%	
**Bell's disability scale***	10-20	0	0	11	4
	30-40	0	0	28	8
	50-60	1	0	6	2
	70-80	2	0	0	1
	90-100	27	15	0	0
**SF-36***	Physical Component Summary **(PCS)**	55.8 +/- 4.3	56.3 +/- 5.2	27.9 +/- 6.8	28.8 +/- 8.6
	Mental Component Summary **(MCS)**	54.7 +/- 5.6	55.8 +/- 4.1	44.1 +/- 10.9	44.5 +/- 8.1

* Higher scores represent better health.
NA: Not applicable.
ND: Not determined.

Supplementary Table 3. Percentages of unknown metabolites for each timepoint and each cohort. Percentages in bold highlight an enrichment of unknowns compared to the percentages of unknowns in the dataset (20%). The information in parenthesis is also visually depicted in Figure 3 as the bars within the histogram bars.

	***q* < 0.05**			***q* < 0.15**			***p* < 0.05**		
	**TOTAL**	**FEMALES**	**MALES**	**TOTAL**	**FEMALES**	**MALES**	**TOTAL**	**FEMALES**	**MALES**
**D1PRE**	**45%** (9/20)	**43%** (3/7)	-	**23%** (13/56)	**22%** (16/72)	-	17% (33/194)	19% (33/174)	**23%** (11/48)
**D1POST**	18% (6/34)	**38%** (9/24)	-	15% (13/88)	**29%** (16/55)	-	16% (32/202)	**22%** (41/185)	**24%** (11/45)
**D2PRE**	14% (8/56)	**27%** (8/30)	-	18% (18/102)	**21%** (23/109)	-	19% (41/211)	19% (41/211)	**24%** (19/78)
**D2POST**	17% (12/69)	**25%** (14/56)	-	15% (23/152)	**20%** (28/143)	-	15% (36/246)	**21%** (49/234)	**28%** (20/72)
